# Transfer of scarlet fever-associated elements into the group A *Streptococcus* M1T1 clone

**DOI:** 10.1038/srep15877

**Published:** 2015-11-02

**Authors:** Nouri L. Ben Zakour, Mark R. Davies, Yuanhai You, Jonathan H. K. Chen, Brian M. Forde, Mitchell Stanton-Cook, Ruifu Yang, Yujun Cui, Timothy C. Barnett, Carola Venturini, Cheryl-lynn Y. Ong, Herman Tse, Gordon Dougan, Jianzhong Zhang, Kwok-Yung Yuen, Scott A. Beatson, Mark J. Walker

**Affiliations:** 1 Australian Infectious Diseases Research Centre, School of Chemistry and Molecular Biosciences, The University of Queensland, Brisbane, QLD 4072, Australia; 2 The Wellcome Trust Sanger Institute, Hinxton, Cambridge, United Kingdom; 3 State Key Laboratory of Infectious Disease Prevention and Control, National Institute for Communicable Disease Control and Prevention, Chinese Center for Disease Control and Prevention, Beijing 102206, China; 4 Collaborative Innovation Center for Diagnosis and Treatment of Infectious Diseases, Hangzhou 310003, Zhejiang, China; 5 Department of Microbiology, The University of Hong Kong, Hong Kong Special Administrative Region, China; 6 Research Centre of Infection and Immunology, The University of Hong Kong, Hong Kong Special Administrative Region, China; 7 State Key Laboratory for Emerging Infectious Diseases, The University of Hong Kong, Hong Kong Special Administrative Region, China; 8 State Key Laboratory of Pathogen and Biosecurity, Beijing Institute of Microbiology and Epidemiology, Beijing 100071, China

## Abstract

The group A *Streptococcus* (GAS) M1T1 clone emerged in the 1980s as a leading cause of epidemic invasive infections worldwide, including necrotizing fasciitis and toxic shock syndrome^1^,^2^,^3^. Horizontal transfer of mobile genetic elements has played a central role in the evolution of the M1T1 clone^4^,^5^, with bacteriophage-encoded determinants DNase Sda1^6^ and superantigen SpeA2^7^ contributing to enhanced virulence and colonization respectively. Outbreaks of scarlet fever in Hong Kong and China in 2011, caused primarily by *emm*12 GAS^8^,^9^,^10^, led to our investigation of the next most common cause of scarlet fever, *emm*1 GAS^8^,^9^. Genomic analysis of 18 *emm*1 isolates from Hong Kong and 16 *emm*1 isolates from mainland China revealed the presence of mobile genetic elements associated with the expansion of *emm*12 scarlet fever clones^10^,^11^ in the M1T1 genomic background. These mobile genetic elements confer expression of superantigens SSA and SpeC, and resistance to tetracycline, erythromycin and clindamycin. Horizontal transfer of mobile DNA conferring multi-drug resistance and expression of a new superantigen repertoire in the M1T1 clone should trigger heightened public health awareness for the global dissemination of these genetic elements.

The bacterial pathogen group A *Streptococcus* (GAS; *Streptococcus pyogenes*) causes over 700 million cases of superficial infections such as pharyngitis and impetigo each year. GAS can also cause severe diseases such as invasive infections, toxic shock, rheumatic heart disease and streptococcal glomerulonephritis, resulting in >500,000 deaths per annum^3^,^12^. Scarlet fever is thought to occur following pharyngeal infection by GAS strains that secrete superantigens, and symptoms include a finely papular deep red rash, “strawberry tongue”, and exudative pharyngitis^13^. While a major cause of childhood morbidity and mortality in the 19^th^ and early 20^th^ centuries, scarlet fever has been in decline as a public health threat for over 100 years^3^,^14^. Beginning in 2011, outbreaks of scarlet fever occurred in mainland China^8^ and Hong Kong^9^,^10^, which are ongoing (Supplementary Fig. 1). Molecular *emm*-typing determined the most common cause of scarlet fever as *emm*12 (serotype M12) GAS^8^,^9^,^10^. Genomic analysis of 144 *emm*12 isolates revealed the multiclonality of outbreak strains, and the association of these strains with the acquisition of integrative and conjugative elements (ICE) encoding resistance to tetracycline, erythromycin and clindamycin, and bacteriophage encoding superantigens SSA and SpeC^11^. Molecular type *emm*1 GAS (serotype M1) was identified as the next most common cause of scarlet fever in mainland China and Hong Kong^8^,^9^. Worldwide, the most frequently isolated GAS strain from severe invasive infections is the M1T1 clone^15^. The M1T1 clone is molecularly typed as *emm*1 and has horizontally acquired a 36 kb *emm*12 chromosomal region encoding toxins NAD-glycohydrolase and streptolysin O, and bacteriophage encoding virulence determinants DNase (Sda1/SdaD2) and the SpeA2 superantigen^1^,^2^,^5^,^15^. The bacteriophage-encoded DNase Sda1 contributes to increased virulence^6^ and superantigen SpeA has been recently demonstrated to enhance colonization^7^. The association of *emm*1 GAS strains with scarlet fever in mainland China and Hong Kong led us to investigate the penetrance of horizontally acquired scarlet fever-associated bacteriophage and ICE elements into the *emm*1 genomic background.

## Results

**Figure 1:**
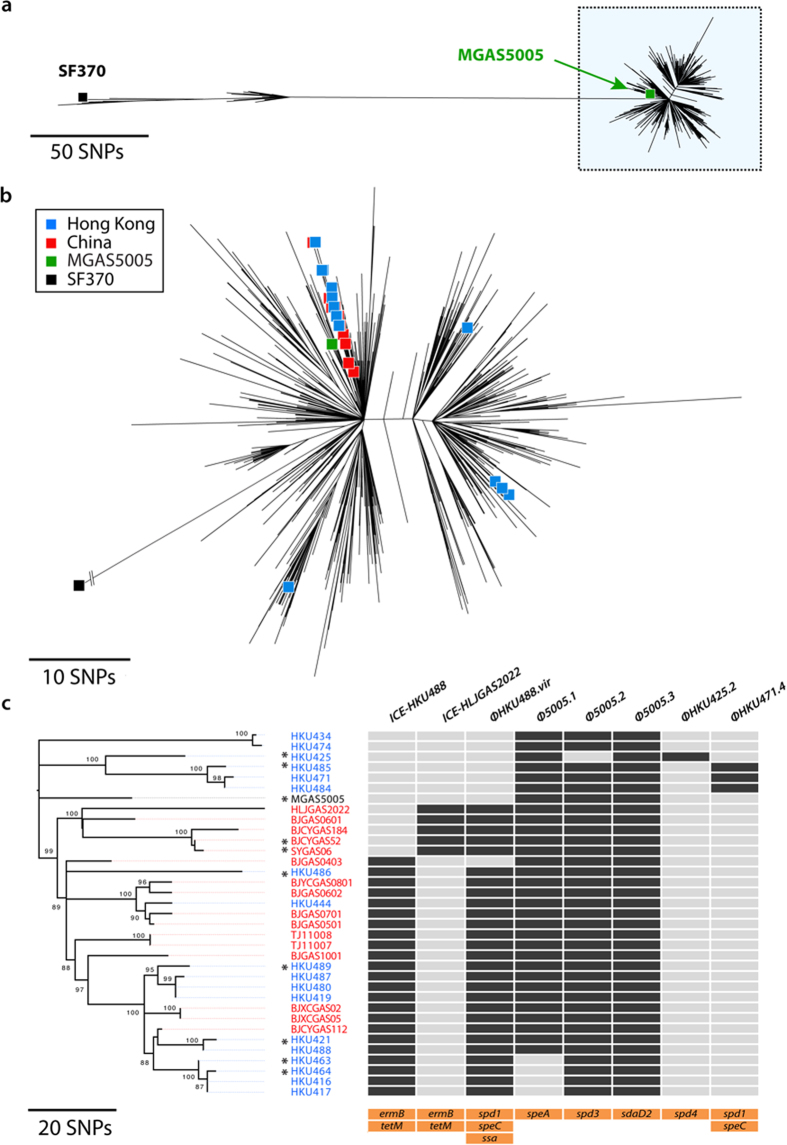
Phylogenetic analysis of *emm*1 GAS. (**a**) Unrooted maximum likelihood phylogenetic tree based on 6,496 core substitutions identified in a global collection of 3,185 *emm*1 strains, including 18 *emm*1 strains from Hong Kong and 16 *emm*1 strains from mainland China, as well as SF370 (black square) and MGAS5005 (green square) highlighted for reference. The blue rectangle on the right of the tree denotes MGAS5005-like strains. (**b**) Genetic relationships of the 3,059 MGAS5005-like strains. The 18 *emm*1 strains from Hong Kong (blue) and 16 *emm*1 strains from mainland China (red), as well as the reference strain MGAS5005 are colored according to their respective position in the tree. (**c**) Maximum likelihood phylogenetic tree based on 417 core substitutions identified in 18 Hong Kong *emm*1 (blue), 16 mainland China *emm*1 (blue) and the MGAS5005 reference strain, rooted to the SF370 reference genome (root not shown). The distribution of mobile genetic elements (bacteriophage ΦHKU488.vir, Φ5005.1, Φ5005.2, Φ5005.3, ΦHKU425.2 and ΦHKU471.4 and integrative conjugative elements ICE-HKU488 and ICE-HLJGAS2022), relative to the phylogenetic tree are shown (presence in dark grey). The bottom three bars shown in orange indicate the various antibiotic resistance and virulence factors associated with each mobile genetic element. GAS isolated from cases other than scarlet fever are denoted with a black asterisk.

We sequenced the genomes of 34 available Hong Kong and mainland China *emm*1 GAS isolates, including 25 confirmed scarlet fever isolates and 9 isolates from other clinical cases (Supplementary Table 1). Next, we performed phylogenetic analyses to investigate clonal relationships between Hong Kong and mainland China *emm*1 GAS isolates and a comprehensive collection of 3,185 *emm*1 strains^4^ representative of the known diversity of the *emm*1 clone. On the basis of 6,496 nucleotide substitutions identified by reference-free *k*-mer based approach within the core *emm*1 genome, all 34 Hong Kong and mainland China *emm*1 GAS isolates cluster with the M1T1 reference strain MGAS5005 (Fig. 1a). The *emm*1 strains from Hong Kong are distributed within 4 separate sub-clades while the *emm*1 strains from mainland China belong to a single clade (Fig. 1b). Temporal regression analysis of the 34 *emm*1 isolates from Hong Kong and mainland China estimated the origin of these clades during the early 1980s (Supplementary Fig. 2). These observations support previous findings suggesting a global expansion of the M1T1 lineage during the 1980s^1^,^2^,^3^,^4^. Of 417 mapping-based core substitutions identified in the 34 *emm*1 strains from Hong Kong and mainland China, there were no substitutions uniquely associated with clinical cases of scarlet fever (Supplementary Table 2).

**Figure 2:**
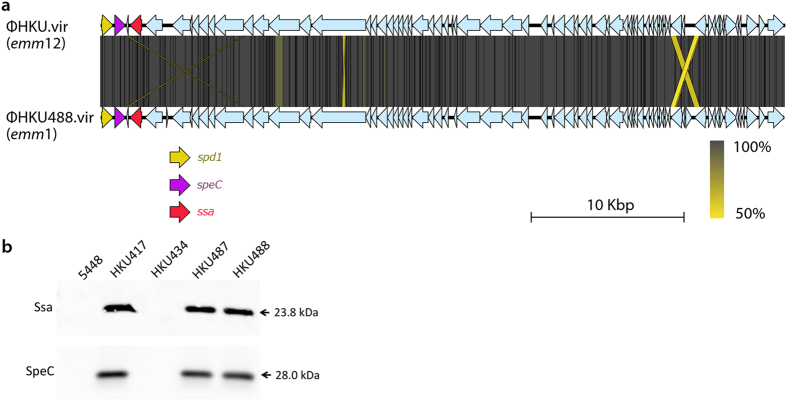
Bacteriophage encoding superantigens SSA and SpeC identified in M1T1 GAS. (**a**) Genetic organization of ΦHKU.vir from HKU16 (*emm*12)^11^ and ΦHKU488.vir from HKU488 (*emm*1). Virulence factors *spd1, speC* and *ssa* are given as yellow, purple, and red arrows, respectively. All other bacteriophage open reading frames are indicated by light blue arrows. Nucleotide sequence identity is graded from 100% (dark grey) to 50% (yellow). Black lines indicate matching tBLASTx block boundaries. (**b**) Western immunoblot detection of SpeC and SSA expression from culture supernatants of representative GAS *emm*1 strains. Expression of SpeC and SSA by GAS strains following overnight growth in THY broth (SpeC blot) or chemically-defined medium (SSA blot). The molecular mass of each protein (kDa) is indicated to the right.

The M1T1 clone is a leading cause of GAS infections worldwide^1^,^2^,^3^. GAS *emm*1 is a significant cause of morbidity in Hong Kong, with *emm*1 GAS replacing *emm*12 GAS as the dominant clinical strain at a major teaching hospital (Queen Mary Hospital) over the 2011-2014 period (Supplementary Table 3). The acquisition of mobile genetic elements such as bacteriophage and integrative conjugative elements can rapidly alter pathogen epidemiology and evolution, and the role of toxin-harboring prophage in the evolution and emergence of GAS is well recognized^16^. The M1T1 clone displays stability in prophage content, with the vast majority (2,903 of 3,443 MGAS5005-like isolates) containing an identical profile consisting of three phage encoding SpeA2, Sda1/SdaD2 and DNase Spd3 respectively^4^. Examination of the prophage content of *emm*1 GAS revealed that 25 of the 34 Hong Kong and mainland China strains harbored prophage ΦHKU488.vir, including 20 of 25 scarlet fever isolates (Fig. 1c). ΦHKU488.vir is a homolog of ΦHKU.vir, a prophage associated with the emergence of scarlet fever *emm*12 GAS clones^11^. Comparison of the sequences of ΦHKU.vir (*emm*12 reference strain HKU16)^10^,^11^ and ΦHKU488.vir (HKU488) revealed that these share 99.9% nucleotide sequence identity, with the key virulence determinant genes *ssa, speC* and *spd1* sharing 100% identity (Fig. 2a). ΦHKU488.vir expresses both the superantigens SSA and SpeC (Fig. 2b). The prophage integration site near the promoter of *uvrA* is identical in HKU16, HKU488 and the other *emm*1 GAS encoding ΦHKU488.vir (Supplementary Fig. 3). These findings strongly suggest horizontal transmission of SSA, SpeC and Spd1 encoding phage between *emm*12 and *emm*1, or from an independent donor. While directionality cannot be inferred with any accuracy, these findings highlight the promiscuity of GAS bacteriophage^16^. Other bacteriophage encoding Spd1 and SpeC (Fig. 1c and Supplementary Fig. 4) and DNases Spd3 and Spd4 (Fig. 1c and Supplementary Fig. 5) are also variably distributed. Scarlet fever is a toxin-mediated disease^3^,^13^ and superantigens SSA, SpeA and SpeC are over-represented in scarlet fever GAS isolates elsewhere^17^. It is striking that 20 of 25 Hong Kong and mainland China *emm*1 scarlet fever isolates contain ΦHKU488.vir integrated into the M1T1 genome, whereas our bioinformatic screening detected *ssa* in less than 0.5% of MGAS5005-like contemporary GAS *emm1* isolates sequenced in a recent study^4^.

**Figure 3:**
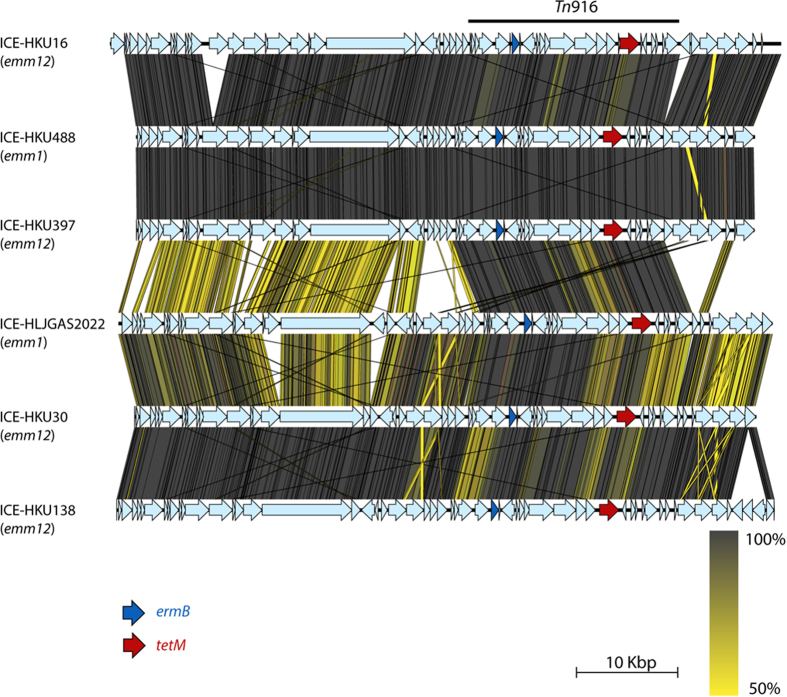
Integrative conjugative elements (ICE) encoding tetracycline and macrolide resistance identified in *emm*1 GAS and comparison to their *emm*12 homologues. Genetic organization of ICE-HKU488 (*emm*1) and ICE-HLJGAS2022 (*emm*12) are compared to ICE-HKU16 (*emm*12)^10^, ICE-HKU397 (*emm*12) and ICE-HKU30 (*emm*12)^11^. Antibiotic resistance genes *ermB* (dark blue arrow) and *tetM* (red arrow), confer resistance to macrolides and tetracycline respectively. All other ICE open reading frames are indicated by light blue arrows. Nucleotide sequence identity is graded from 100% (dark grey) to 50% (yellow). Black lines indicate matching tBLASTx block boundaries.

An alarming feature of GAS epidemiology in Hong Kong and mainland China has been the high levels of resistance to macrolides (erythromycin, azithromycin, and clarithromycin), the lincosamide antibiotic clindamycin, and tetracycline^10^,^11^,^18^. This multidrug resistance is mediated by integrative and conjugative elements (ICE) encoding both macrolide (*ermB*) and tetracycline (*tetM*) resistance^10^,^11^. Less than 0.5% of 3,443 previously examined MGAS5005-like strains encode *ermB*^4^. In contrast, 23 of the 34 Hong Kong and mainland China *emm*1 isolates harbored an ICE element encoding *ermB* and *tetM*, herein defined as ICE-HKU488, which shared 99.9% and 99.5% nucleotide sequence identity with the *emm*12 ICE-HKU397 and ICE-HKU16 respectively (Fig. 3). The ICE integration site is identical in HKU16, HKU397, HKU488 and the other *emm*1 GAS, and is located at the 3′ end of a tRNA uracil methyltransferase gene (Supplementary Fig. 3). An additional 5 strains from mainland China harbor a 64 Kb ICE-HKU30-like^11^ element designated ICE-HLJGAS2022. This element also encodes *ermB* and *tetM*, and, similar to ICE-HKU30, appears to be located in a distinct genomic location from ICE-HKU488 (Fig. 3 and Supplementary Fig. 3). The acquisition of both *ermB* and *tetM* encoding ICE and the ΦHKU488.vir phage occurs in a single *emm*1 sub-clade and is retained by all strains once acquired (Fig. 1c).

## Discussion

Between 2011 and 2012, >100,000 scarlet fever cases in mainland China were reported by the Chinese Ministry of Health. Since September 2013, Public Health England have reported a scarlet fever outbreak of >15,000 cases. The evolutionary forces driving these outbreaks are currently unknown, but bacterial determinants (strain replacement, virulence gene acquisition), host immune status and environmental factors (such as temperature and rainfall) may all play a significant role^19^. The results of this current study are deeply concerning for a number of reasons. Firstly, the M1T1 clone emerged in the 1980s and disseminated as a global health threat^1^,^2^,^3^,^4^,^5^,^15^. Acquisition of new superantigens by the M1T1 clone, including the scarlet fever-associated superantigen SSA^11^,^17^, has the potential to change the pathogenesis and disease association of these strains, and underlines the fundamentally important role bacteriophage play in GAS evolution^16^. Only heightened surveillance and epidemiological analyses will determine the full impact these gene acquisitions will have on global GAS disease burden, though it is notable in this study that multiple *emm*1 scarlet fever isolates encode SSA. Secondly, prescription of macrolides and clindamycin is common in primary health care as a broad spectrum treatment for respiratory tract infections^19^. Acquisition of macrolide resistance into the M1T1 genomic background will likely present a further challenge at the primary health care level for the treatment of such infections. The use of penicillin in non-allergic patients remains an excellent treatment alternative for GAS infections, as all strains remain penicillin sensitive^3^.

## Methods

### GAS *emm*1 strain collection

Clinical GAS isolates were typed as *emm*1 according to standard procedures described by the Centre for Disease Control and Prevention ( http://www.cdc.gov/streplab/M-ProteinGene-typing.html). A total of 34 *emm*1 isolates from Hong Kong and mainland China, including 25 from scarlet fever diagnosed patients, were characterized. Antibiotic sensitivity was determined as previously defined^10^,^11^. Clinical and molecular characteristics of the isolates are listed in Supplementary Table 1. Genomic DNA was extracted from 37 °C overnight 1.8 ml brain heart infusion broth cultures using the DNeasy Tissue Kit (Qiagen). To place the Hong Kong and mainland China *emm*1 GAS examined in this study within the global evolutionary context of the *emm*1 GAS clone, the sequencing data of the most temporally and geographically comprehensive collection of 3,615 *emm*1 strains previously published^4^ was retrieved for these analyses.

### Genome sequencing and comparative genomics analysis

Paired-end multiplex libraries were created as described previously^20^ followed by sequencing on the Illumina Hi-seq 2000 platform, with a read length set at 100 bp. Illumina sequence reads for the Hong Kong and mainland China *emm*1 collections were deposited in the European Nucleotide Archive under the accession codes listed in Supplementary Table 1. Prior to use, quality control (QC) checks were performed to ensure a mean base-pair quality score of Q> = 20 and a read length > = 85 bp, for a post-QC average coverage ranging from 97–140×. HKU488 was selected as a representative strain and subjected to PacBio (Pacific Biosciences) sequencing to confirm the sequence of complex elements such as prophage and ICE identified in *emm*1 scarlet fever isolates. PacBio sequencing was generated on the Pacific Biosciences RS II platform from a single molecule real-time (SMRT) cell as previously described^11^. *De novo* assembly of HKU488 was performed using PacBio’s SMRT Portal (v2.2.0) and the hierarchical genome assembly process HGAP^21^. The final assembly obtained reached an estimated average coverage of 114×. *De novo* assembly of Illumina sequencing data for the 34 *emm*1 strains was performed using Velvet 1.2.07^22^. All assemblies were then annotated using Prokka 1.10^23^. Comparative genomic analyses between individual strains were performed using a combination of tools, namely BLASTn, tBLASTx^24^, Artemis^25^ and Easyfig^26^.

### Phylogenetic analysis

*De novo* draft genome assemblies from a collection of 3,615 *emm*1 strains^4^ were generated with SPAdes 3.0.0^27^ using raw reads. Due to the majority of this dataset consisting of 42 bp single-end Illumina reads, a total of 432 assemblies associated with either low sequence coverage <20×, highly fragmented assembly (>1000 contigs) or contaminated samples were discarded from the analysis. A final set of draft genomes comprising 3,185 *emm*1 strains (including SF370 and MGAS5005) as well as the 34 *emm*1 Hong Kong and mainland China strains was then used as an input to determine genome-wide core SNPs using the reference-free *k*-mer based approach implemented in kSNP 2.1.2^28^, resulting in a 6,496 bp core-SNP matrix. To avoid the detrimental impact of sub-optimal assemblies on core SNPs estimation, we performed high-resolution phylogenetic inference using a mapping approach, where reads from each of the 34 Hong Kong and mainland China *emm*1 strains were mapped against the MGAS5005 reference genome using SHRIMP 2.0^29^. Nesoni 0.108 ( www.vicbioinformatics.com/software.nesoni.shtml) was then used to perform SNP calling (set on default parameters) as well as predict coding-effect SNP annotation. A 417 core SNP matrix was identified by performing *n-way* pairwise comparison as implemented in Nesoni, and discarding SNPs associated with mobile genetic elements.

For the phylogeny reconstruction analysis, we estimated maximum likelihood trees using RAxML 7.2.8^30^ under the GTR nucleotide substitution model with a gamma correction for ASRV, for both the 3,219 global *emm*1 strains 6,496 bp core SNP matrix and the 34 *emm*1 Hong Kong and mainland China only 417 core SNP matrix, assessing node support using 100 and 1000 random bootstrap replicates, respectively. To estimate the underlying temporal signal of the 34 Hong Kong and mainland China *emm*1 strains and MGAS5005, we used Path-O-Gen v1.4 ( http://www.tree.bio.ed.ac.uk/software/pathogen), which performs a regression analysis between the genetic distance calculated from the root to the tips of the previously estimated 417 SNP-based phylogeny and the year of isolation of each strain.

### Bioinformatic screening for *ssa*

Screening for the presence of *ssa* was performed using SRST2 using both single-end and paired-end raw reads and default parameters^31^.

### SSA and SpeC western blots

Primary antibodies used were rabbit anti-SpeC (ab16024; Abcam) and affinity-purified rabbit antisera (produced by Mimotopes, Clayton, Australia) raised against the peptide H-CGGSSQPDPTPEQLNKSSQFTG-OH coupled to Keyhole Limpet Hemocyanin (anti-SSA). Overnight cultures of GAS strains were grown in THY broth containing 28 uM of the cysteine proatease inhibitor E64, or modified chemically defined medium (Gibco RPMI 1640, no glucose (Life Technologies) supplemented with 1% D-Glucose, 3.2 mM L-cysteine, and components 2 (amino acids), 3 (vitamins) and 5 (nucleobases) of the GAS chemically defined medium, pH 7.5). GAS supernatants were precipitated with 10% TCA and each protein was detected by western immunoblot, as described previously^11^.

## Additional Information

**How to cite this article**: Ben Zakour, N. L. *et al.* Transfer of scarlet fever-associated elements into the group A *Streptococcus* M1T1 clone. *Sci. Rep.* **5**, 15877; doi: 10.1038/srep15877 (2015).

## Supplementary Material

Supplementary Information
