# Supplementary material for: Transfer of scarlet fever-associated elements into the group A Streptococcus M1T1 clone
Source: Sci Rep. 2015 Nov 2;5:15877. doi: 10.1038/srep15877 (PMC4629146; doi:10.1038/srep15877)
Supplement: Supplementary Information [file srep15877-s1.pdf]

Transfer of scarlet fever-associated elements into the group A *Streptococcus* M1T1 clone

Nouri L. Ben Zakour[1,^], Mark R. Davies[1,2,^], Yuanhai You[3,4], Jonathan H. K. Chen[5,6,7], Brian Forde[1], Mitchell Stanton-Cook[1], Ruifu Yang[8], Yujun Cui[8], Timothy C. Barnett[1], Carola Venturini[1], Cheryl-lynn Y. Ong[1], Herman Tse[5,6,7], Gordon Dougan[2,#], Jianzhong Zhang[3,4,#], Kwok-Yung Yuen[5,6,7,#], Scott A. Beatson[1,#,*], Mark J. Walker[1,#,*]

[1]Australian Infectious Diseases Research Centre, School of Chemistry and Molecular Biosciences, The University of Queensland, Brisbane, QLD 4072, Australia.

[2]The Wellcome Trust Sanger Institute, Hinxton, Cambridge, United Kingdom.

State Key Laboratory for Infectious Disease Prevention and Control, National Institute for Communicable Disease

[3]Control and Prevention, Chinese Center for Disease Control and Prevention, Beijing 102206, China.

[4]Collaborative Innovation Center for Diagnosis and Treatment of Infectious Diseases, Hangzhou 310003, Zhejiang, China.

[5]Department of Microbiology, The University of Hong Kong, Hong Kong Special Administrative Region, China.

[6]Research Centre of Infection and Immunology, The University of Hong Kong, Hong Kong Special Administrative Region, China.

[7]State Key Laboratory for Emerging Infectious Diseases, The University of Hong Kong, Hong Kong Special Administrative Region, China.

[8]State Key Laboratory of Pathogen and Biosecurity, Beijing Institute of Microbiology and Epidemiology, Beijing 100071, China

SUPPLEMENTARY FIGURE LEGENDS

Supplementary Figure 1. Clinical cases of scarlet fever in Hong Kong and mainland China. Monthly notifications (black bars) of scarlet fever cases reported in Hong Kong (**a**) by the Centre for Health Protection and Beijing (**b**) by the Chinese Centre for Disease Control since 2007. The dashed red line represents monthly rainfall data for Hong Kong and Beijing.

Supplementary Figure 2. Temporal analysis of *emm*1 GAS from Hong Kong and mainland China. Linear regression correlation plot derived from the root-to-tip branch lengths extracted from the maximum-likelihood tree (Figure 1c) and the year of strain isolation as estimated using Path-O-Gen (http://tree.bio.ed.ac.uk/software/pathogen/) for 34 *emm*1 GAS from Hong Kong (blue) and mainland China (red) strains, and the reference strain MGAS5005 (black).

Supplementary Figure 3. Whole genome comparison of representative *emm*1 GAS strains. Prophage and ICE are indicated by rectangles, colored according to sequence similarity as follows: ICE-*emm*12-like (yellow), ΦHKU.vir-like (green), ΦHKU370.1-like (olive), Φ5005.1-like (light blue), Φ5005.2-like (red), Φ5005.2 variants ΦHKU425.2 and ΦSF370.2 (light red), Φ5005.3-like (mauve), Φ9429.2 and Φ370.3 (brown). Nucleotide sequence identity is graded from 100% (dark grey) to 68% (light grey), being the minimum value observed for pairwise matches that could be depicted. Black lines indicate matching BLASTn block boundaries.

Supplementary Figure 4. Genetic organization of ΦHKU471.4 compared to 4 closely related prophage: ΦSF370.1 (*emm*1), ΦMGAS2096.1 (*emm*12), HKU160.1 (*emm*12) and ΦMGAS9429 (*emm*12). Virulence factors *spd1* and *speC* are shown in yellow and purple

respectively. All other bacteriophage open reading frames are indicated by light blue arrows. Nucleotide sequence identity is graded from 100% (dark grey) to 50% (yellow). Black lines indicate matching tBLASTx block boundaries. Red line indicates contig boundaries.

Supplementary Figure 5. Genetic organization of ΦHKU425.2 compared to 3 closely related prophages: ΦMGAS5005.2 (*emm*1), HKU488.2 (*emm*1) and ΦMGAS315.3 (*emm*3). Virulence factors *spd3* and *spd4* are shown in dark blue and green respectively. All other bacteriophage open reading frames are indicated by light blue arrows. Nucleotide sequence identity is graded from 100% (dark grey) to 50% (yellow). Black lines indicate matching tBLASTx block boundaries.

Supplementary Table 1. Mainland China and Hong Kong *emm*1 GAS strains sequenced in this study.

Supplementary Table 2. Single nucleotide polymorphisms identified in 34 *emm*1 strains from mainland China and Hong Kong relative to the MGAS5005 reference genome.

Supplementary Table 3. Distribution of GAS *emm* types from clinical cases presenting at Queen Mary Hospital, Hong Kong (2011-2014).

a

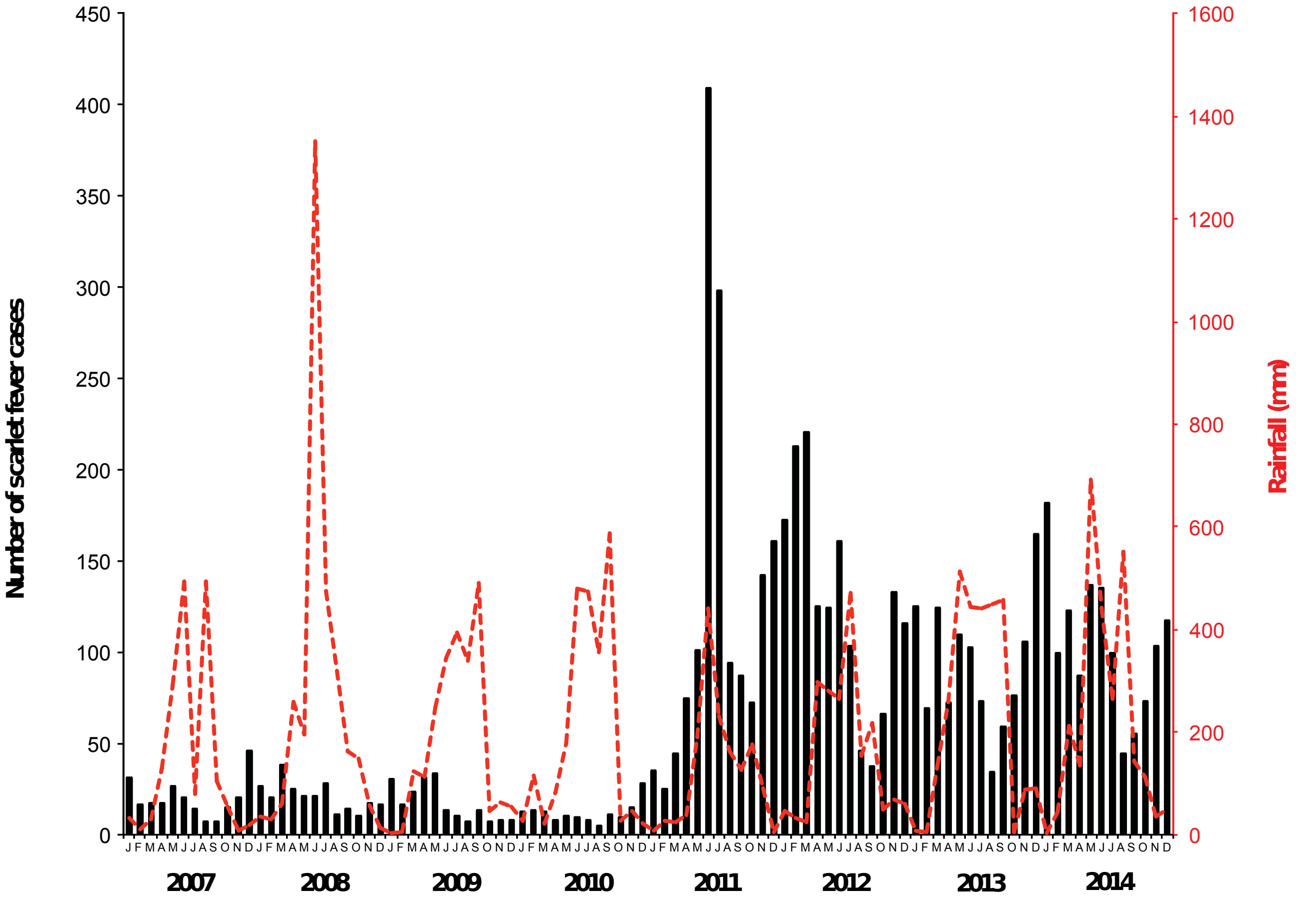

Number of scarlet fever cases
0
50
100
150
200
250
300
350
400
450
Rainfall (mm)
0
200
400
600
800
1000
1200
1400
1600
2007
2008
2009
2010
2011
2012
2013
2014

b

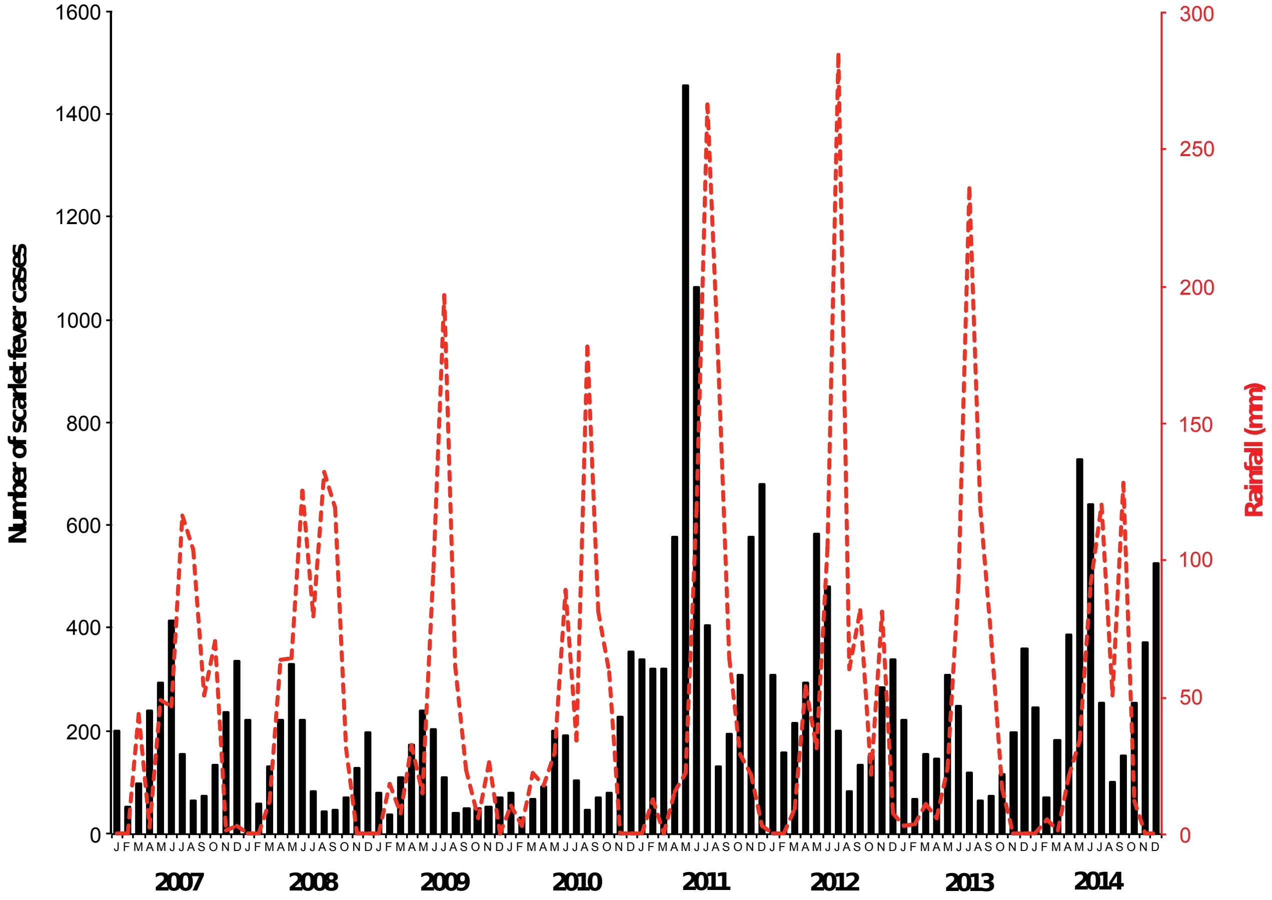

Number of scarlet fever cases
0
200
400
600
800
1000
1200
1400
1600
Rainfall (mm)
0
50
100
150
200
250
300
2007
2008
2009
2010
2011
2012
2013
2014

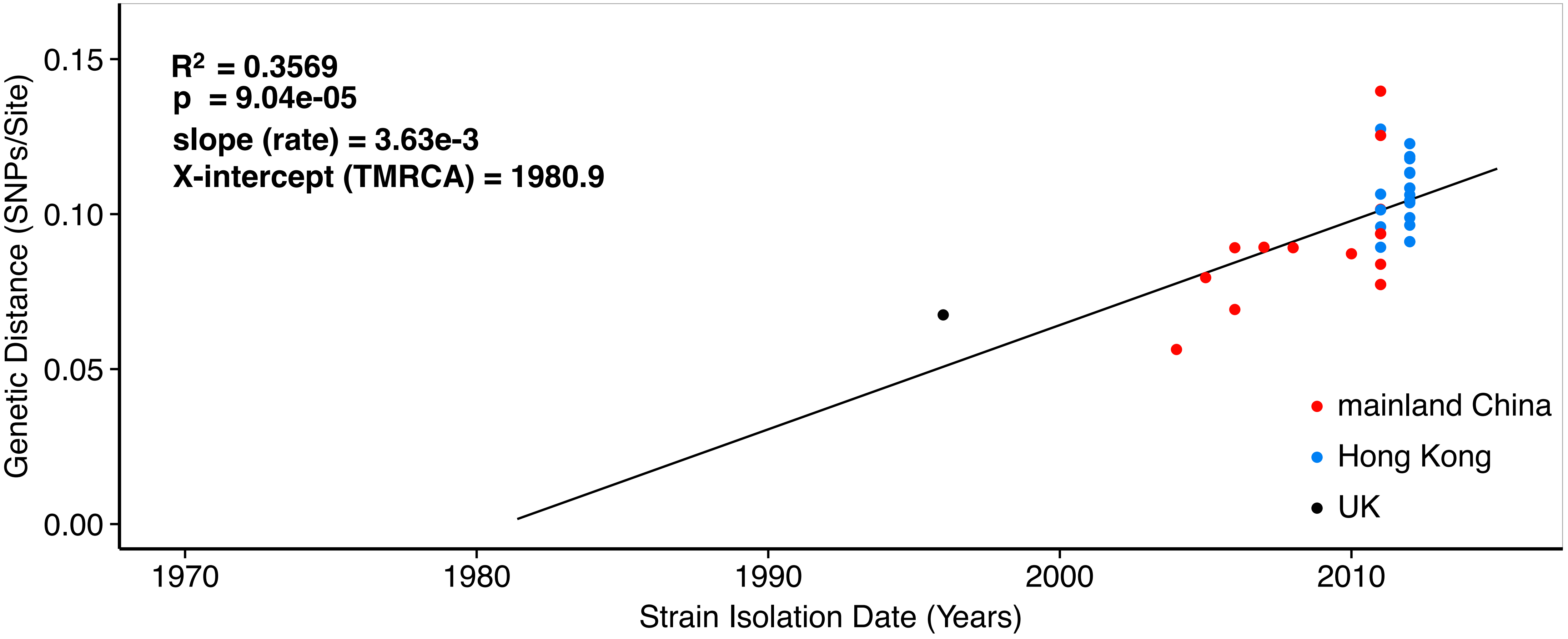

R^2 = 0.3569
p = 9.04e-05
slope (rate) = 3.63e-3
X-intercept (TMRCA) = 1980.9
0.15
0.10
0.05
0.00
Genetic Distance (SNPs/Site)
1970
1980
1990
2000
2010
Strain Isolation Date (Years)
mainland China
Hong Kong
UK

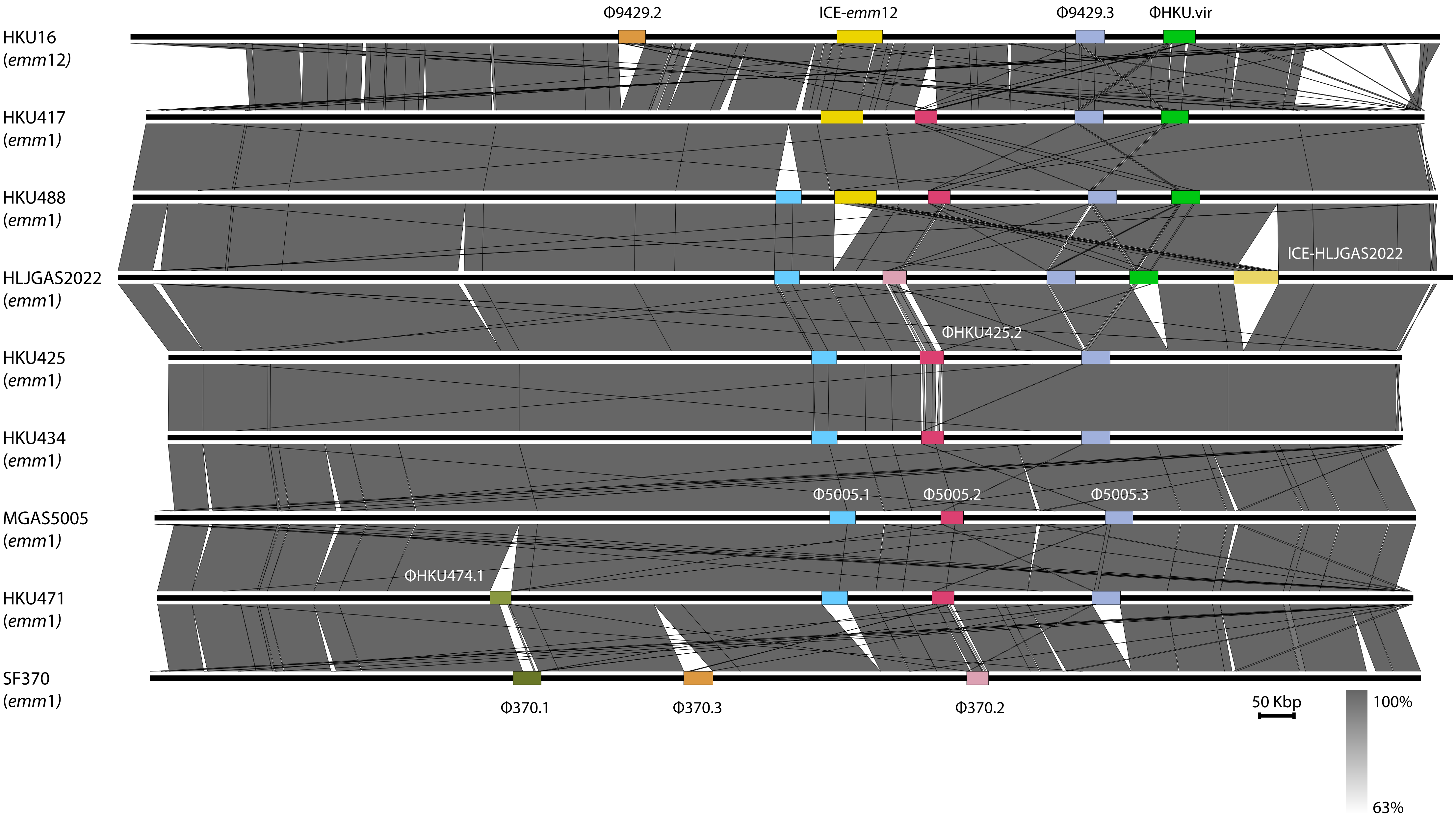

Φ9429.2
ICE-emm12
Φ9429.3
ΦHKU.vir
HKU16
(emm12)
HKU417
(emm1)
HKU488
(emm1)
ICE-HLJGAS2022
HLJGAS2022
(emm1)
ΦHKU425.2
HKU425
(emm1)
HKU434
(emm1)
Φ5005.1
Φ5005.2
Φ5005.3
MGAS5005
(emm1)
ΦHKU474.1
HKU471
(emm1)
SF370
(emm1)
Φ370.1
Φ370.3
Φ370.2
50 Kbp
100%
63%

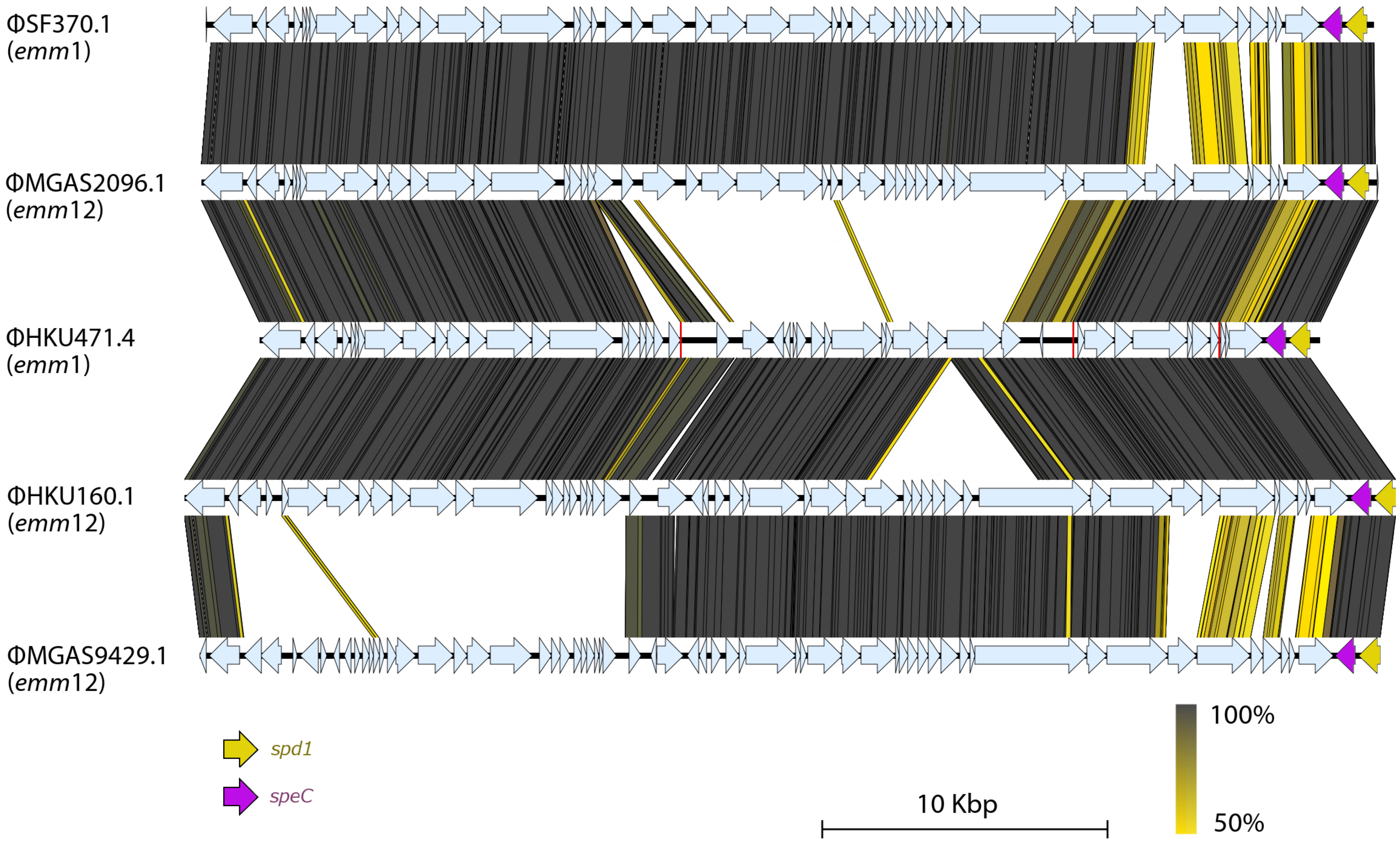
ΦSF370.1
(emm1)
ΦMGAS2096.1
(emm12)
ΦHKU471.4
(emm1)
ΦHKU160.1
(emm12)
ΦMGAS9429.1
(emm12)
spd1
speC
10 Kbp
100%
50%

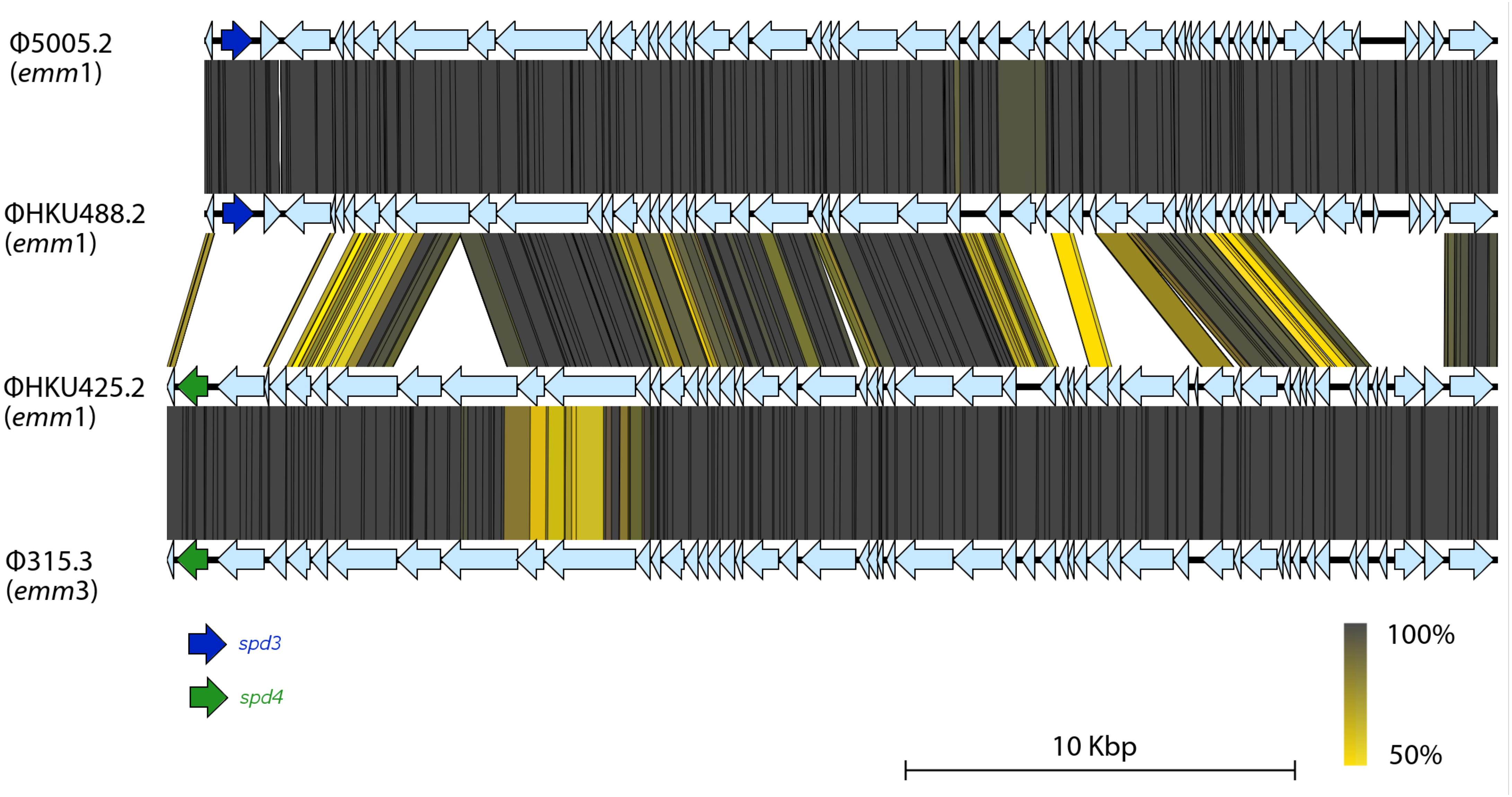
Φ5005.2
(emm1)
ΦHKU488.2
(emm1)
ΦHKU425.2
(emm1)
Φ315.3
(emm3)
spd3
spd4
10 Kbp
100%
50%

Supplementary Table 1. Mainland China and Hong Kong *emm*1 GAS strains sequenced in this study.

Strain number	Isolation date	Resistance	Patient Age (y)	Specimen	Clinical presentation	Country of origin	Accession no.	Molecular screening												
								tetM	*ermB*	*ssa*	*speC*	*sda1*	*spd1*	*spd3*	*spd4*	*speA*	*speG*	*speJ*	*smeZ*	*speB*
BJCYGAS112	2011	Ery/CLD/TET	3	Throat swab	Scarlatina	mainland China	TBC	+	+	+	+	+	+	+	-	+	+	+	+	+
BJCYGAS184	2011	Ery/CLD/TET	11	Throat swab	Scarlatina	mainland China	TBC	+	+	+	+	+	+	+	-	+	+	+	+	+
BJCYGAS52	2011	Ery/CLD/TET	13	Throat swab	Pharyngitis	mainland China	TBC	+	+	+	+	+	+	+	-	+	+	+	+	+
BJGAS0403	2004	NA	<15	Throat swab	Scarlatina	mainland China	TBC	+	+	-	-	+	-	+	-	+	+	+	+	+
BJGAS0501	2005	NA	<15	Throat swab	Scarlatina	mainland China	TBC	+	+	+	+	+	+	+	-	+	+	+	+	+
BJGAS0601	2006	NA	<15	Throat swab	Scarlatina	mainland China	TBC	+	+	+	+	+	+	+	-	+	+	+	+	+
BJGAS0602	2006	NA	<15	Throat swab	Scarlatina	mainland China	TBC	+	+	+	+	+	+	+	-	+	+	+	+	+
BJGAS0701	2007	NA	<15	Throat swab	Scarlatina	mainland China	TBC	+	+	+	+	+	+	+	-	+	+	+	+	+
BJGAS1001	2010	NA	<15	Throat swab	Scarlatina	mainland China	TBC	+	+	+	+	+	+	+	-	+	+	+	+	+
BJXCGAS02	2011	NA	9	Throat swab	Scarlatina	mainland China	TBC	+	+	+	+	+	+	+	-	+	+	+	+	+
BJXCGAS05	2011	NA	4	Throat swab	Scarlatina	mainland China	TBC	+	+	+	+	+	+	+	-	+	+	+	+	+
BJYCGAS-0801	2008	NA	<15	Throat swab	Scarlatina	mainland China	TBC	+	+	+	+	+	+	+	-	+	+	+	+	+
HKU416	18/01/12	Ery/CLD/TET	14	Pleural fluid	Scarlet fever	Hong Kong	ERR172161	+	+	+	+	+	+	+	-	-	+	+	+	+
HKU417	20/01/12	Ery/CLD/TET	11	Blood culture	Scarlet fever	Hong Kong	ERR172162	+	+	+	+	+	+	+	-	-	+	+	+	+
HKU419	23/02/12	Ery/CLD/TET	8	Blood culture	Scarlet fever	Hong Kong	ERR172163	+	+	+	+	+	+	+	-	+	+	+	+	+
HKU421	1/02/12	Ery/CLD/TET	3	Throat swab	Rash	Hong Kong	ERR172164	+	+	+	+	+	+	+	-	+	+	+	+	+
HKU425	11/01/12	ND	48	Left anterior arm deep fasciitis tissue	Necrotizing fasciitis	Hong Kong	ERR172165	-	-	-	-	+	-	-	+	+	+	+	+	+
HKU434	2/07/11	ND	6	Throat swab	Scarlet fever	Hong Kong	ERR172166	-	-	-	-	+	-	+	-	+	+	+	+	+
HKU444	21/07/11	Ery/CLD/TET	6	Throat swab	Scarlet fever	Hong Kong	ERR172167	+	+	+	+	+	+	+	-	+	+	+	+	+
HKU463	10/01/12	Ery/CLD/TET	5	Throat swab	Upper respiratory tract infection	Hong Kong	ERR172168	+	+	+	+	+	+	+	-	-	+	+	+	+
HKU464	11/01/12	Ery/CLD/TET	76	Blood culture	Pneumonia	Hong Kong	ERR172169	+	+	+	+	+	+	+	-	-	+	+	+	+
HKU471	29/01/12	ND	4	Throat swab	Scarlet fever	Hong Kong	ERR172170	-	-	-	+	+	+	+	-	+	+	+	+	+
HKU474	6/02/12	ND	12	Throat swab	Scarlet fever	Hong Kong	ERR172171	-	-	-	-	+	-	+	-	+	+	+	+	+
HKU480	19/02/12	Ery/CLD/TET	8	Throat swab	Scarlet fever, necrotizing pneumonia	Hong Kong	ERR172172	+	+	+	+	+	+	+	-	+	+	+	+	+
HKU484	27/02/12	ND	7	Throat swab	Scarlet fever	Hong Kong	ERR172173	-	-	-	+	+	+	+	-	+	+	+	+	+
HKU485	1/03/12	ND	63	Blood culture	Fever, left chronic suppurative otitis media	Hong Kong	ERR172174	-	-	-	+	+	+	+	-	+	+	+	+	+
HKU486	19/11/11	Ery/CLD/TET	6	Throat swab	Fever	Hong Kong	ERR172175	+	+	+	+	+	+	+	-	+	+	+	+	+
HKU487	21/11/11	Ery/CLD/TET	10	Throat swab	Scarlet fever	Hong Kong	ERR172176	+	+	+	+	+	+	+	-	+	+	+	+	+
HKU488	8/01/12	Ery/CLD/TET	8	Throat swab	Scarlet fever	Hong Kong	ERR172177	+	+	+	+	+	+	+	-	+	+	+	+	+
HKU489	5/02/12	Ery/CLD/TET	10	Throat swab	Henoch-Schönlein purpura	Hong Kong	ERR172178	+	+	+	+	+	+	+	-	+	+	+	+	+
HLJGAS2022	2011	Ery/CLD/TET	7	Throat swab	Scarlatina	mainland China	TBC	+	+	+	+	+	+	+	-	+	+	+	+	+
SYGAS06	2011	NA	11	Throat swab	NA	mainland China	TBC	+	+	+	+	+	+	+	-	+	+	+	+	+
TJ11-007	2011	Ery/CLD/TET	4	Throat swab	Scarlatina	mainland China	TBC	+	+	+	+	+	+	+	-	+	+	+	+	+
TJ11-008	2011	Ery/CLD/TET	9	Throat swab	Scarlatina	mainland China	TBC	+	+	+	+	+	+	+	-	+	+	+	+	+

Abbreviations

ND	None detected
NA	Not available
Ery	Erythromycin resistant
CLD	Clindamycin resistant
TET	Tetracycline resistant
+	gene present
-	gene absent
TBC	To be confirmed

Supplementary Table 2. Single nucleotide polymorphisms identified in 34 *emm*1 strains from mainland China and Hong Kong relative to the MGAS5005 reference genome.

Position	Change type	MGAS5005	BJCYGAS112	BJCYGAS184	BJCYGAS52	BJGAS0403	BJGAS0501	BJGAS0701	BJGAS0601	BJGAS1001	BJGAS0602	BJXCGAS02	BJXCGAS05	BJYCGAS0801	HLJGAS2022	SYGAS06	TJ11007	TJ11008	HKU416	HKU417	HKU419	HKU421	HKU425	HKU434	HKU444	HKU463	HKU464	HKU471	HKU474	HKU480	HKU484	HKU485	HKU486	HKU487	HKU488	HKU489	SNP effect	Locus_tag	Gene	Base	Codon	Product
9875	substitution	C	C	C	C	C	C	C	C	C	C	C	C	C	C	C	C	C	C	C	C	C	C	T	C	C	C	C	T	C	C	C	C	C	C	C	P=>L	M5005_Spy_0010		239	80	beta-lactamase
10941	substitution	T	T	C	C	T	T	T	C	T	T	T	T	T	C	C	T	T	T	T	T	T	T	T	T	T	T	T	T	T	T	T	T	T	T	T	F=>L	M5005_Spy_0011	*tilS*	22	8	tRNA(Ile)-lysidine synthetase
12192	substitution	T	A	T	T	T	T	T	T	T	T	A	A	T	T	T	T	T	A	A	A	A	T	T	T	A	A	T	T	A	T	T	T	A	A	A	L=>I	M5005_Spy_0011	*tilS*	1273	425	tRNA(Ile)-lysidine synthetase
16844	substitution	A	A	A	A	A	A	A	A	G	A	A	A	A	A	A	A	A	A	A	A	A	A	A	A	A	A	A	A	A	A	A	A	A	A	A	synonymous	M5005_Spy_0015		102	34	hypothetical protein
30833	substitution	A	A	A	A	G	A	A	A	A	A	A	A	A	A	A	A	A	A	A	A	A	A	A	A	A	A	A	A	A	A	A	A	A	A	A						
33536	substitution	G	G	G	G	G	G	G	G	G	G	G	G	G	G	G	G	G	G	G	G	G	A	G	G	G	G	A	G	G	A	A	G	G	G	G	M=>I	M5005_Spy_0018	*prsA.2*	954	318	ribose-phosphate pyrophosphokinase
35892	substitution	A	A	A	A	A	A	A	A	A	A	A	A	A	T	A	A	A	A	A	A	A	A	A	A	A	A	A	A	A	A	A	A	A	A	A						
39663	substitution	C	C	C	C	C	C	C	C	C	C	C	C	C	C	C	C	C	C	C	C	C	C	C	C	C	C	T	C	C	T	T	C	C	C	C	Q=>*	M5005_Spy_0023		2902	968	phosphoribosylformylglycinamidine synthase
41716	substitution	C	C	T	C	C	C	C	C	C	C	C	C	C	C	C	C	C	C	C	C	C	C	C	C	C	C	C	C	C	C	C	C	C	C	C	synonymous	M5005_Spy_0024	*purF*	1021	341	amidophosphoribosyltransferase
48755	substitution	G	C	C	C	C	C	C	C	C	C	C	C	C	C	C	C	C	C	C	C	C	G	G	C	C	C	G	G	C	G	G	C	C	C	C	R=>P	M5005_Spy_0030	*purE*	245	82	phosphoribosylaminoimidazole carboxylase catalytic subunit
48972	substitution	C	C	T	T	C	C	C	C	C	C	C	C	C	C	T	C	C	C	C	C	C	C	C	C	C	C	C	C	C	C	C	C	C	C	C	synonymous	M5005_Spy_0030	*purE*	462	154	phosphoribosylaminoimidazole carboxylase catalytic subunit
50038	substitution	A	A	A	A	A	A	A	A	A	A	A	A	A	A	A	A	A	A	A	A	A	A	A	A	A	A	A	A	A	A	A	G	A	A	A	synonymous	M5005_Spy_0031	*purK*	933	311	phosphoribosylaminoimidazole carboxylase ATPase subunit
51337	substitution	C	C	C	C	C	C	C	C	C	C	C	C	C	C	C	C	C	C	C	C	C	C	C	C	C	C	C	C	C	C	C	T	C	C	C	R=>C	M5005_Spy_0032		1129	377	hypothetical protein
69368	substitution	C	C	C	C	C	C	C	C	C	C	C	C	C	C	C	C	C	C	C	C	C	C	C	C	C	C	C	C	C	C	C	T	C	C	C	T=>I	M5005_Spy_0053	*rpsQ*	23	8	30S ribosomal protein S17
73743	substitution	C	C	C	C	C	C	C	C	C	C	C	C	C	C	C	C	C	C	C	C	C	C	T	C	C	C	C	T	C	C	C	C	C	C	C						
90914	substitution	G	G	A	G	G	G	G	G	G	G	G	G	G	G	G	G	G	G	G	G	G	G	G	G	G	G	G	G	G	G	G	G	G	G	G	T=>I	M5005_Spy_0081	*tyrS*	560	187	tyrosyl-tRNA synthetase
94071	substitution	T	T	T	T	T	C	C	T	T	C	T	T	C	T	T	T	T	T	T	T	T	T	T	C	T	T	T	T	T	T	T	T	T	T	T						
98982	substitution	C	C	C	C	C	C	C	C	C	C	C	C	C	C	C	C	C	C	C	C	C	C	T	C	C	C	C	T	C	C	C	C	C	C	C	synonymous	M5005_Spy_0084	*rpoC*	1185	395	DNA-directed RNA polymerase subunit beta'
99317	substitution	A	A	A	A	A	A	A	A	A	A	A	A	A	A	A	A	A	A	A	A	A	A	A	A	A	A	A	A	A	A	A	G	A	A	A	D=>G	M5005_Spy_0084	*rpoC*	1520	507	DNA-directed RNA polymerase subunit beta'
99969	substitution	G	G	G	G	G	G	G	G	G	G	G	G	G	G	G	G	G	G	G	G	G	G	C	G	G	G	G	C	G	G	G	G	G	G	G	K=>N	M5005_Spy_0084	*rpoC*	2172	724	DNA-directed RNA polymerase subunit beta'
106289	substitution	G	G	G	G	G	G	G	G	A	G	G	G	G	G	G	G	G	G	G	G	G	G	G	G	G	G	G	G	G	G	G	G	G	G	G	E=>K	M5005_Spy_0093		526	176	adenine-specific methyltransferase
111923	substitution	G	G	G	G	G	G	G	G	G	G	G	G	G	G	G	G	G	G	G	G	G	A	G	G	G	G	A	G	G	A	A	G	G	G	G	synonymous	M5005_Spy_0101		408	136	tRNA-binding domain-containing protein
115616	substitution	C	C	C	C	C	C	C	C	C	C	C	C	C	C	C	C	C	C	C	C	C	T	C	C	C	C	C	C	C	C	C	C	C	C	C	D=>N	M5005_Spy_0106	*rofA*	1471	491	transcriptional regulator
123668	substitution	G	G	G	G	G	T	T	G	G	T	G	G	T	G	G	G	G	G	G	G	G	G	G	T	G	G	G	G	G	G	G	G	G	G	G	S=>Y	M5005_Spy_0112		461	154	transposase
126433	substitution	T	T	T	T	T	T	T	T	T	T	T	T	T	T	T	T	T	T	T	T	T	T	T	T	T	C	T	T	T	T	T	T	T	T	T	T=>A	M5005_Spy_0115		205	69	hypothetical protein
130650	substitution	G	G	G	G	G	G	G	G	G	G	G	G	G	G	G	G	G	G	G	G	G	G	A	G	G	G	G	A	G	G	G	G	G	G	G	G=>D	M5005_Spy_0119		1160	387	acetyl-CoA acetyltransferase
132902	substitution	G	G	G	G	G	G	G	G	G	G	G	G	G	G	G	G	G	G	G	G	G	T	G	G	G	G	G	G	G	G	G	G	G	G	G						
140764	substitution	G	G	G	G	G	G	G	G	G	G	G	G	G	G	G	G	G	G	G	G	G	A	G	G	G	G	G	G	G	G	G	G	G	G	G	synonymous	M5005_Spy_0131	*ntpA*	921	307	V-type ATP synthase subunit A
141608	substitution	G	G	G	G	G	G	G	G	G	G	G	G	G	G	G	G	G	G	G	G	G	G	G	G	G	G	G	G	G	G	G	A	G	G	G	G=>R	M5005_Spy_0131	*ntpA*	1765	589	V-type ATP synthase subunit A
152621	substitution	A	A	A	A	A	A	A	A	A	A	A	A	A	A	A	A	A	A	A	A	A	A	G	A	A	A	A	G	A	A	A	A	A	A	A	synonymous	M5005_Spy_0141	*slo*	591	197	streptolysin O
154454	substitution	C	C	C	C	C	C	C	C	C	C	C	C	C	C	C	T	T	C	C	C	C	C	C	C	C	C	C	C	C	C	C	C	C	C	C						
154911	substitution	C	C	C	C	C	C	C	C	C	C	C	C	C	C	C	C	C	C	C	C	C	C	C	C	C	C	C	C	C	C	C	C	C	C	A						
158809	substitution	T	T	T	T	T	T	T	T	T	T	T	T	T	T	T	T	T	T	T	T	T	T	T	T	T	T	C	T	T	C	C	T	T	T	T	I=>T	M5005_Spy_0147	*leuS*	1073	358	leucyl-tRNA synthetase
167459	substitution	C	C	C	C	C	C	C	C	C	C	C	C	C	C	C	C	C	C	C	C	C	T	C	C	C	C	T	C	C	T	T	C	C	C	C						
168776	substitution	T	T	T	T	T	T	T	T	C	T	T	T	T	T	T	T	T	T	T	T	T	T	T	T	T	T	T	T	T	T	T	T	T	T	T						
173704	substitution	G	A	G	G	G	G	G	G	G	G	G	G	G	G	G	G	G	G	G	G	A	G	G	G	G	G	G	G	G	G	G	G	G	A	G	A=>T	M5005_Spy_0159	*polA*	1570	524	DNA polymerase I
178952	substitution	T	T	T	T	T	T	T	T	T	T	T	T	T	T	T	T	T	T	T	T	T	T	T	T	T	T	T	T	T	T	C	T	T	T	T	synonymous	M5005_Spy_0166		66	22	transposase
179216	substitution	A	C	A	A	A	A	A	A	A	A	C	C	A	A	A	A	A	C	C	C	C	A	A	A	C	C	A	A	C	A	A	A	C	C	C	F=>L	M5005_Spy_0167		411	137	transposase
179754	substitution	C	T	C	C	C	C	C	C	C	C	T	T	C	C	C	C	C	T	T	T	T	C	C	C	T	T	C	C	T	C	C	C	T	T	T	E=>K	M5005_Spy_0168		121	41	transposase
192082	substitution	C	C	C	C	C	C	C	C	C	C	C	C	C	C	C	C	C	C	C	C	C	C	T	C	C	C	C	T	C	C	C	C	C	C	C						
195849	substitution	G	G	G	G	G	G	G	G	G	G	G	G	G	A	G	G	G	G	G	G	G	G	G	G	G	G	G	G	G	G	G	G	G	G	G						
195914	substitution	T	T	C	C	T	T	T	T	T	T	T	T	T	T	C	T	T	T	T	T	T	T	T	T	T	T	T	T	T	T	T	T	T	T	T						
197179	substitution	T	C	C	C	C	C	C	C	C	C	C	C	C	C	C	C	C	C	C	C	C	C	C	C	C	C	C	C	C	C	C	C	C	C	C	V=>A	M5005_Spy_0189		14	5	hypothetical protein
197315	substitution	G	G	G	G	G	G	G	G	G	G	G	G	G	T	G	G	G	G	G	G	G	G	G	G	G	G	G	G	G	G	G	G	G	G	G						
202237	substitution	G	G	G	G	G	G	G	G	G	G	G	G	G	G	G	G	G	G	G	A	G	G	G	G	G	G	G	G	A	G	G	G	A	G	G	G=>S	M5005_Spy_0196		1273	425	multidrug resistance ABC transporter ATP-binding protein/permease
204027	substitution	G	G	G	G	G	G	G	G	G	G	G	G	G	G	G	G	G	G	G	G	G	G	G	G	G	G	A	G	G	A	G	G	G	G	G	G=>S	M5005_Spy_0197		1354	452	multidrug resistance ABC transporter ATP-binding protein/permease
209011	substitution	C	C	C	C	C	C	C	C	C	C	C	C	C	C	C	C	C	C	C	C	C	C	C	C	C	C	C	C	C	C	C	T	C	C	C						
211052	substitution	C	C	C	C	C	C	C	C	C	C	C	C	C	C	C	C	C	C	C	C	C	C	T	C	C	C	C	T	C	C	C	C	C	C	C	P=>S	M5005_Spy_0204	*fasB*	163	55	sensory transduction protein kinase
217529	substitution	C	C	C	C	C	C	C	A	C	C	C	C	C	C	C	C	C	C	C	C	C	C	C	C	C	C	C	C	C	C	C	C	C	C	C						
223515	substitution	C	C	C	C	C	C	C	C	C	C	C	C	C	C	C	T	T	C	C	C	C	C	C	C	C	C	C	C	C	C	C	C	C	C	C	synonymous	M5005_Spy_0218		243	81	N-acetylmannosamine kinase
225506	substitution	G	G	G	G	G	G	G	G	G	G	G	G	G	G	G	G	G	G	G	G	G	G	G	G	G	G	A	G	G	A	A	G	G	G	G	G=>R	M5005_Spy_0220	*tatD*	103	35	sec-independent protein translocase
234026	substitution	T	T	T	T	T	T	T	T	T	T	T	T	T	C	T	T	T	T	T	T	T	T	T	T	T	T	T	T	T	T	T	T	T	T	T	synonymous	M5005_Spy_0229	*prgA*	360	120	surface exclusion protein
234479	substitution	T	T	C	C	T	T	T	T	T	T	T	T	T	T	C	T	T	T	T	T	T	T	T	T	T	T	T	T	T	T	T	T	T	T	T	synonymous	M5005_Spy_0229	*prgA*	813	271	surface exclusion protein
236290	substitution	G	G	A	A	G	G	G	G	G	G	G	G	G	G	A	G	G	G	G	G	G	G	G	G	G	G	G	G	G	G	G	G	G	G	G						
243667	substitution	T	T	T	T	T	T	T	T	A	T	T	T	T	T	T	T	T	T	T	T	T	T	T	T	T	T	T	T	T	T	T	T	T	T	T	T=>S	M5005_Spy_0236		322	108	amino acid ABC transporter permease
243672	substitution	C	C	C	C	C	C	C	C	C	C	T	T	C	C	C	C	C	C	C	C	C	C	C	C	C	C	C	C	C	C	C	C	C	C	C	S=>N	M5005_Spy_0236		317	106	amino acid ABC transporter permease
271393	substitution	T	C	T	T	T	T	T	T	C	T	C	C	T	T	T	C	C	C	C	C	C	T	T	T	C	C	T	T	C	T	T	T	C	C	C						
271530	substitution	C	C	C	C	C	C	C	C	C	C	C	C	C	C	C	A	A	C	C	C	A	C	C	C	C	C	C	C	C	C	C	C	C	A	C	S=>Y	M5005_Spy_0257		74	25	transposase
277385	substitution	T	T	T	T	T	T	T	T	T	T	T	T	T	T	T	T	T	T	T	T	T	T	T	T	T	T	T	T	T	T	T	A	T	T	T						
279154	substitution	C	C	T	C	C	C	C	C	C	C	C	C	C	C	C	C	C	C	C	C	C	C	C	C	C	C	C	C	C	C	C	C	C	C	C	T=>I	M5005_Spy_0269		134	45	hypothetical protein
281002	substitution	A	A	A	A	A	A	A	A	C	A	A	A	A	A	A	A	A	A	A	A	A	A	A	A	A	A	A	A	A	A	A	A	A	A	A						
282343	substitution	C	C	C	C	C	T	T	C	C	T	C	C	T	C	C	C	C	C	C	C	C	C	C	T	C	C	C	C	C	C	C	C	C	C	C	synonymous	M5005_Spy_0272		138	46	ABC transporter ATP-binding protein
284257	substitution	T	C	C	C	C	C	C	C	C	C	C	C	C	C	C	C	C	C	C	C	C	T	T	C	C	C	T	T	C	T	T	C	C	C	C	synonymous	M5005_Spy_0274	*braB*	1131	377	branched-chain amino acid transporter carrier protein
289022	substitution	C	C	C	C	C	C	T	C	C	C	C	C	C	C	C	C	C	C	C	C	C	C	C	C	C	C	C	C	C	C	C	C	C	C	C						
291767	substitution	C	C	C	C	C	C	C	C	C	C	C	C	C	C	C	C	C	C	C	C	C	T	C	C	C	C	T	C	C	T	T	C	C	C	C	R=>C	M5005_Spy_0281		136	46	hypothetical protein
293788	substitution	C	C	C	C	C	C	C	C	C	C	C	C	C	C	C	C	C	C	C	C	C	C	T	C	C	C	C	T	C	C	C	C	C	C	C	synonymous	M5005_Spy_0283	*covS*	528	176	transmembrane histidine kinase
300522	substitution	C	C	C	C	C	C	C	C	C	C	C	C	C	C	C	C	C	C	C	C	C	C	T	C	C	C	C	T	C	C	C	C	C	C	C	T=>I	M5005_Spy_0288	*snf*	1547	516	SWF/SNF family helicase
310166	substitution	T	T	T	T	T	T	T	T	T	T	T	T	T	T	T	T	T	T	T	T	T	T	T	T	T	T	T	T	T	T	G	T	T	T	T	Y=>S	M5005_Spy_0298		74	25	transposase
313961	substitution	T	T	C	T	T	T	T	T	T	T	T	T	T	T	T	T	T	T	T	T	T	T	T	T	T	T	T	T	T	T	T	T	T	T	T	M=>T	M5005_Spy_0304		56	19	deoxyribonucleotide triphosphate pyrophosphatase/unknown domain fusion protein
316551	substitution	G	G	G	G	G	G	G	G	G	A	G	G	A	G	G	G	G	G	G	G	G	G	G	G	G	G	G	G	G	G	G	G	G	G	G	synonymous	M5005_Spy_0307	*xerD*	705	235	site-specific tyrosine recombinase XerD
337668	substitution	G	G	G	G	G	G	G	G	G	G	G	G	G	G	G	G	G	G	G	G	G	G	G	G	G	G	G	G	G	G	G	A	G	G	G	synonymous	M5005_Spy_0331	*dnaX*	12	4	DNA polymerase III subunit delta'
338180	substitution	A	G	G	G	G	G	G	G	G	G	G	G	G	G	G	G	G	G	G	G	G	G	G	G	G	G	G	G	G	G	G	G	G	G	G	E=>G	M5005_Spy_0331	*dnaX*	524	175	DNA polymerase III subunit delta'
343226	substitution	C	C	C	C	C	C	C	C	C	C	C	C	C	C	C	C	C	C	C	C	C	C	T	C	C	C	C	T	C	C	C	C	C	C	C						
344029	substitution	G	G	G	G	G	G	G	G	G	G	G	G	G	G	G	G	G	G	G	G	G	G	A	G	G	G	G	A	G	G	G	G	G	G	G	synonymous	M5005_Spy_0340	*lctO*	759	253	L-lactate oxidase
345806	substitution	A	A	A	A	A	A	A	A	A	A	A	A	A	G	A	A	A	A	A	A	A	A	A	A	A	A	A	A	A	A	A	A	A	A	A	synonymous	M5005_Spy_0341		1092	364	lactocepin
347531	substitution	T	T	T	T	T	T	T	T	T	T	T	T	T	T	T	T	T	T	T	T	T	T	T	C	T	T	T	T	T	T	T	T	T	T	T	synonymous	M5005_Spy_0341		2817	939	lactocepin
348654	substitution	T	T	T	T	T	T	T	T	T	T	T	T	T	G	T	T	T	T	T	T	T	T	T	T	T	T	T	T	T	T	T	T	T	T	T	S=>A	M5005_Spy_0341		3940	1314	lactocepin
348788	substitution	T	T	T	T	T	G	G	T	T	G	T	T	G	T	T	T	T	T	T	T	T	T	T	G	T	T	T	T	T	T	T	T	T	T	T	synonymous	M5005_Spy_0341		4074	1358	lactocepin
354317	substitution	G	G	G	G	G	G	G	G	G	G	G	G	G	G	G	G	G	G	G	G	G	G	A	G	G	G	G	A	G	G	G	G	G	G	G	synonymous	M5005_Spy_0347	*nrdF*	447	149	ribonucleotide-diphosphate reductase subunit beta
355116	substitution	C	C	C	C	C	C	C	C	C	C	C	C	C	C	C	C	C	C	C	C	C	T	C	C	C	C	C	C	C	C	C	C	C	C	C	H=>Y	M5005_Spy_0348	*nrdI*	229	77	ribonucleotide reductase stimulatory protein
358563	substitution	G	G	G	G	G	G	A	G	G	G	G	G	G	G	G	G	G	G	G	G	G	G	G	G	G	G	G	G	G	G	G	G	G	G	G						
360427	substitution	T	T	T	T	T	T	T	T	T	T	T	T	T	T	T	T	T	T	T	T	T	T	T	T	T	T	T	T	T	T	T	C	T	T	T	T=>A	M5005_Spy_0354		298	100	hypothetical protein
360729	substitution	T	T	T	T	T	T	T	T	T	T	T	T	T	T	T	T	T	T	T	T	T	T	T	T	T	T	T	T	T	T	T	C	T	T	T						
363296	substitution	A	A	A	A	A	A	A	A	A	A	A	A	A	A	A	A	A	A	A	A	A	A	A	A	A	G	A	A	A	A	A	A	A	A	A	Y=>H	M5005_Spy_0357		301	101	hypothetical protein
367693	substitution	G	A	G	G	G	G	G	G	G	G	A	A	G	G	G	G	G	A	A	A	A	G	G	G	A	A	G	G	A	G	G	G	A	A	A						
370576	substitution	C	C	C	C	C	C	C	C	C	C	C	C	C	C	C	C	C	C	C	C	C	C	T	C	C	C	C	T	C	C	C	C	C	C	C	R=>C	M5005_Spy_0365	*pfs*	577	193	5'-methylthioadenosine/S-adenosylhomocysteine nucleosidase
377980	substitution	C	C	C	C	T	C	C	C	C	C	C	C	C	C	C	C	C	C	C	C	C	C	C	C	C	C	C	C	C	C	C	C	C	C	C	A=>V	M5005_Spy_0372	*ftsK*	2153	718	cell division protein
389380	substitution	A	A	A	A	A	A	A	A	A	A	A	A	A	A	A	A	A	A	A	A	A	G	A	A	A	A	A	A	A	A	A	A	A	A	A	D=>G	M5005_Spy_0386	*phoH*	968	323	phoH protein
393827	substitution	T	T	C	C	T	T	T	C	T	T	T	T	T	C	C	T	T	T	T	T	T	T	T	T	T	T	T	T	T	T	T	T	T	T	T	synonymous	M5005_Spy_0393		229	77	hypothetical protein
399088	substitution	A	A	A	A	A	A	A	A	A	A	A	A	A	G	A	A	A	A	A	A	A	A	A	A	A	A	A	A	A	A	A	A	A	A	A						
405044	substitution	C	C	T	C	C	C	C	C	C	C	C	C	C	C	C	C	C	C	C	C	C	C	C	C	C	C	C	C	C	C	C	C	C	C	C	P=>L	M5005_Spy_0411		956	319	multidrug resistance protein B
405727	substitution	T	T	T	T	T	T	T	T	T	T	T	T	T	T	T	T	T	T	T	T	C	T	T	T	T	T	T	T	T	T	T	T	T	C	T						
409274	substitution	G	G	G	G	G	G	G	G	G	G	G	G	G	G	G	G	G	G	G	G	G	G	G	G	G	G	G	A	G	G	G	G	G	G	G	R=>H	M5005_Spy_0416		656	219	glutaminyl-peptide cyclotransferase
416354	substitution	C	C	T	T	C	C	C	C	C	C	C	C	C	C	T	C	C	C	C	C	C	C	C	C	C	C	C	C	C	C	C	C	C	C	C	L=>F	M5005_Spy_0424	*ccpA*	961	321	catabolite control protein A
416966	substitution	G	G	G	G	G	G	G	G	G	G	G	G	G	T	G	G	G	G	G	G	G	G	G	G	G	G	G	G	G	G	G	G	G	G	G	K=>N	M5005_Spy_0425		441	147	glycosyltransferase
433589	substitution	T	T	T	T	T	T	T	T	C	T	T	T	T	T	T	T	T	T	T	T	T	T	T	T	T	T	T	T	T	T	T	T	T	T	T	V=>A	M5005_Spy_0439	*smc*	1271	424	chromosome partition protein
436669	substitution	T	T	T	T	T	T	T	T	T	T	T	T	T	T	T	T	T	A	A	T	T	T	T	T	A	A	T	T	T	T	T	T	T	T	T	L=>F	M5005_Spy_0440		294	98	transcriptional regulator
439030	substitution	A	A	A	A	A	A	A	A	A	A	G	G	A	A	A	A	A	A	A	A	A	A	A	A	A	A	A	A	A	A	A	A	A	A	A	T=>A	M5005_Spy_0443		88	30	hypothetical protein
456130	substitution	C	C	C	C	C	C	C	C	C	C	C	C	C	C	C	C	C	T	T	C	C	C	C	C	T	T	C	C	C	C	C	C	C	C	C	synonymous	M5005_Spy_0465		156	52	hypothetical protein
460526	substitution	C	C	C	C	C	C	C	C	C	C	C	C	C	C	C	C	C	C	C	C	C	C	C	C	C	C	G	C	C	C	C	C	C	C	C	synonymous	M5005_Spy_0471		738	246	HAD superfamily hydrolase
462989	substitution	G	G	G	G	G	G	G	A	G	G	G	G	G	G	G	G	G	G	G	G	G	G	G	G	G	G	G	G	G	G	G	G	G	G	G	P=>L	M5005_Spy_0473		596	199	multidrug resistance protein B
466506	substitution	A	A	A	A	A	A	A	A	A	A	A	A	A	A	A	A	A	A	A	A	A	A	A	A	A	A	G	A	A	G	G	A	A	A	A	Y=>C	M5005_Spy_0475		1589	530	PTS system beta-glucoside-specific transporter subunit IIABC
467171	substitution	C	C	C	C	C	C	C	C	C	C	C	C	C	C	C	C	C	C	C	C	T	C	C	C	C	C	C	C	C	C	C	C	C	C	C	P=>S	M5005_Spy_0476	*bglA*	535	179	6-phospho-beta-glucosidase
470449	substitution	T	T	T	T	T	T	T	T	T	T	T	T	T	T	T	T	T	T	T	T	T	T	T	T	T	T	T	T	T	T	T	C	T	T	T	synonymous	M5005_Spy_0480		36	12	transcription accessory protein
471283	substitution	C	C	C	C	C	T	T	C	C	T	C	C	T	C	C	C	C	C	C	C	C	C	C	T	C	C	C	C	C	C	C	C	C	C	C	synonymous	M5005_Spy_0480		870	290	transcription accessory protein
481866	substitution	C	C	T	T	C	C	C	T	C	C	C	C	C	C	T	C	C	C	C	C	C	C	C	C	C	C	C	C	C	C	C	C	C	C	C	synonymous	M5005_Spy_0496		231	77	HAD superfamily hydrolase
484215	substitution	G	G	G	G	G	T	T	G	G	T	G	G	T	G	G	G	G	G	G	G	G	G	G	T	G	G	G	G	G	G	G	G	G	G	G	P=>T	M5005_Spy_0499		529	177	thiamine transporter
484385	substitution	C	C	C	C	C	C	C	C	C	C	T	T	C	C	C	C	C	C	C	C	C	C	C	C	C	C	C	C	C	C	C	C	C	C	C	S=>N	M5005_Spy_0499		359	120	thiamine transporter
486321	substitution	C	C	C	C	C	C	C	C	C	C	C	C	C	C	C	C	C	C	C	C	C	C	C	C	C	C	C	C	C	C	C	T	C	C	C	A=>V	M5005_Spy_0501		137	46	hypothetical protein
503206	substitution	T	T	T	T	T	A	A	T	T	A	T	T	A	T	T	T	T	T	T	T	T	T	T	A	T	T	T	T	T	T	T	T	T	T	T	L=>H	M5005_Spy_0516	*pacL*	1070	357	calcium-transporting ATPase
506702	substitution	G	G	G	G	G	G	G	G	G	G	G	G	G	G	G	G	G	G	G	G	G	G	G	A	G	G	G	G	G	G	G	G	G	G	G	synonymous	M5005_Spy_0518		1167	389	oligohyaluronate lyase
518521	substitution	C	C	C	C	C	C	C	C	C	C	C	C	C	C	C	C	C	C	C	C	C	T	C	C	C	C	C	C	C	C	C	C	C	C	C	A=>V	M5005_Spy_0530	*prfB*	416	139	peptide chain release factor 2
531519	substitution	C	C	A	C	C	C	C	C	C	C	C	C	C	C	C	C	C	C	C	C	C	C	C	C	C	C	C	C	C	C	C	C	C	C	C	A=>E	M5005_Spy_0542	*pepD*	5	2	dipeptidase
532494	substitution	C	C	C	C	C	C	C	C	C	C	C	C	C	C	C	C	C	C	C	C	C	T	C	C	C	C	C	C	C	C	C	C	C	C	C	P=>L	M5005_Spy_0542	*pepD*	980	327	dipeptidase
544618	substitution	C	C	C	C	C	C	C	C	C	C	C	C	C	C	C	C	C	C	C	C	C	C	T	C	C	C	C	T	C	C	C	C	C	C	C	synonymous	M5005_Spy_0553	*gyrB*	1914	638	DNA gyrase subunit B
549751	substitution	A	A	A	A	A	A	A	A	A	A	A	A	A	A	A	G	G	A	A	A	A	A	A	A	A	A	A	A	A	A	A	A	A	A	A	L=>P	M5005_Spy_0557		239	80	transposase
559808	substitution	A	A	A	A	A	A	A	A	A	A	A	A	A	A	A	A	A	A	A	A	A	G	A	A	A	A	G	A	A	G	G	A	A	A	A	synonymous	M5005_Spy_0562	*sagA*	102	34	streptolysin S
570249	substitution	C	C	C	C	C	C	C	C	C	C	C	C	C	C	C	C	C	C	C	C	C	T	C	C	C	C	C	C	C	C	C	C	C	C	C	synonymous	M5005_Spy_0571		1683	561	hypothetical protein
571932	substitution	C	C	C	C	C	C	C	C	C	A	C	C	C	C	C	C	C	C	C	C	C	C	C	C	C	C	C	C	C	C	C	C	C	C	C	T=>N	M5005_Spy_0572		356	119	hypothetical protein
577697	substitution	G	G	G	G	G	G	G	G	G	G	G	G	G	G	G	A	A	G	G	G	G	G	G	G	G	G	G	G	G	G	G	G	G	G	G	A=>T	M5005_Spy_0579	*atpA*	28	10	ATP synthase F0F1 subunit alpha

578636	substitution	G	GGGGGGGGGGGGGGGG	GGAGGGGGGGGAGGGAGA	A=>T	M5005_Spy_0579	*atpA*	967	323	ATP synthase F0F1 subunit alpha
582143	substitution	C	CCCCCCCCCCCCCCCC	CCCCCTCCCCTCCCCCCC						
582325	substitution	G	GGGGGGGGGGGGGGGG	GGGGAGGGGGGGGGGGGG						
592919	substitution	A	CCCCCCCCCCCCCCCC	CCCCCCCCCCCCCCCCCC	K=>Q	M5005_Spy_0594	*rexB*	424	142	ATP-dependent nuclease subunit B
597192	substitution	T	TTTTTTTTTTTTTTTT	TTTCTTTTTTTTTTTTCT	synonymous	M5005_Spy_0595	*rexA*	1521	507	ATP-dependent nuclease subunit A
602727	substitution	G	GAAGGGGGGGGGGAGG	GGGGGGGGGGGGGGGGGG	D=>N	M5005_Spy_0599	*dnaG*	1498	500	DNA primase
602861	substitution	C	CCCCCCCCCCCCCCCC	CCCCCCCCCCCCCCACCC	N=>K	M5005_Spy_0599	*dnaG*	1632	544	DNA primase
603458	substitution	G	GGGGGGGGGGGGGGGG	GGGGAGGGGAGGAAGGGG	A=>T	M5005_Spy_0600	*rpoD*	406	136	RNA polymerase sigma factor RpoD
607766	substitution	A	AAAACCAAAAAAAAAA	AAAAAACAAAAAAAAAAA	D=>A	M5005_Spy_0604	*rgpBc*	776	259	alpha-L-Rha alpha-1;3-L-rhamnosyltransferase
608103	substitution	C	CCCCCCCCCCCCCCCC	CCCCCCCCCCCCCCTCCC	P=>S	M5005_Spy_0605	*rgpCc*	178	60	polysaccharide export ABC transporter permease
609673	substitution	C	CCCCCCCCCCCCCCCC	CCCCCCCCCTCCTTCCCC	synonymous	M5005_Spy_0606	*rgpDc*	945	315	polysaccharide export ATP-binding protein
613039	substitution	C	CCCCCCTCCCCCCCCC	CCCCCCCCCCCCCCCCCC	T=>I	M5005_Spy_0609		335	112	phosphoglycerol transferase
613789	substitution	G	GGGGAAGGAGGAGGGG	GGGGGGAGGGGGGGGGGG	R=>Q	M5005_Spy_0609		1085	362	phosphoglycerol transferase
614328	substitution	C	CCCCCCCCCCCCCCCC	CCCCCTCCCCTCCCCCCC	P=>S	M5005_Spy_0609		1624	542	phosphoglycerol transferase
624001	substitution	G	GGGGGGGGAGGAGGGG	GGGGGGGGGGGGGGGGGG						
629758	substitution	G	GGGGGGGAGGGGGGGG	GGGGGGGGGGGGGGGGGG	E=>K	M5005_Spy_0626		232	78	hypothetical protein
630326	substitution	G	GGGGGGAGGGGGGGGG	GGGGGGGGGGGGGGGGGG	D=>N	M5005_Spy_0627	*gor*	238	80	glutathione reductase
632747	substitution	A	AAAAAAAAAAAAAAAA	AAAACAAAAAAAAAAAAA	synonymous	M5005_Spy_0628	*folC.2*	51	17	folylpolyglutamate synthase/dihydrofolate synthase
639130	substitution	A	AAAAAAAAAAAAAAAA	AAAAAAAAAAAAAAAAAG	synonymous	M5005_Spy_0636		396	132	LysR family transcriptional regulator
639397	substitution	C	CCCCCCCCCCCCCCTT	CCCCCCCCCCCCCCCCCC	synonymous	M5005_Spy_0636		663	221	LysR family transcriptional regulator
639916	substitution	G	GGGGGGGGGGGGGGGG	GGGGGAGGGGGGGGGGGG	A=>T	M5005_Spy_0637	*lsp*	271	91	lipoprotein signal peptidase
643368	substitution	T	TTTTTTTTTTTTTCTT	TTTTTTTTTTTTTTTTTT	synonymous	M5005_Spy_0641	*pyrB*	132	44	aspartate carbamoyltransferase
646251	substitution	A	AAAAAAAAAAAAAAAA	AAAAAAAAAAAAAATAAA	H=>L	M5005_Spy_0643	*carB*	728	243	carbamoyl phosphate synthase large subunit
654281	substitution	T	TTTTAATTATTATTTT	TTTTTTATTTTTTTTTTT	synonymous	M5005_Spy_0648	*rpsP*	48	16	30S ribosomal protein S16
660114	substitution	G	GGGGGGGGGGGGAGGG	GGGGGGGGGGGGGGGGGG	L=>F	M5005_Spy_0653	*czcD*	55	19	cobalt-zinc-cadmium resistance protein
669956	substitution	T	TTTTTTTTTTTTCTTT	TTTTTTTTTTTTTTTTTT						
681843	substitution	C	CCCCCTCCCCCCCCCC	CCCCCCCCCCCCCCCCCC	A=>V	M5005_Spy_0679		101	34	GTP pyrophosphokinase
687194	substitution	C	CCCCCCCCCCCCCCCC	CCCCACCCCACCAACCCC	L=>I	M5005_Spy_0684	*mvaK2*	799	267	phosphomevalonate kinase
688685	substitution	G	GGGGGGGGGGGGGGGG	AAGGGGGAAGGGGGGGGG	A=>V	M5005_Spy_0686		1208	403	3-hydroxy-3-methylglutaryl-CoA reductase
702938	substitution	A	GAAAAAAAAGGAAAAA	GGGGAAAGGAAGAAAGGG	N=>S	M5005_Spy_0700	*cpsX*	83	28	LytR family transcriptional regulator
714905	substitution	C	CCCCCCCCCCCCCCCC	CCCCCCCCCTCCTTCCCC	synonymous	M5005_Spy_0711	*parE*	1533	511	DNA topoisomerase IV subunit B
715660	substitution	A	AAAAAAAAAAAAGAAA	AAAAAAAAAAAAAAAAAA	D=>G	M5005_Spy_0712	*parC*	248	83	DNA topoisomerase IV subunit A
718186	substitution	C	CCCTCCCCCCCCCCCC	CCCCCCCCCCCCCCCCCC	synonymous	M5005_Spy_0713	*bcaT*	192	64	branched-chain amino acid aminotransferase
724842	substitution	G	GAAGGGGGGGGGGAGG	GGGGGGGGGGGGGGGGGG						
728255	substitution	A	AAAAAAAAAAACAAAA	AAAAAAAAAAAAAAAAAA	synonymous	M5005_Spy_0725	*elaC*	159	53	ribonuclease Z
731989	substitution	G	GGGGGGGGGGGGGGGG	GGGGGGGGGTGGGGGGGG	D=>Y	M5005_Spy_0727	*recJ*	2203	735	single-stranded-DNA-specific exonuclease
733896	substitution	C	CCCCCCCCCCCCCCCC	TTCCCCCTTCCCCCCCCC	A=>V	M5005_Spy_0730	*nth*	470	157	endonuclease III
737598	substitution	G	GGGGGGGGGGGGGGGG	GGAGGGGGGGGAGGGAGG	G=>D	M5005_Spy_0734	*cpsFO*	776	259	glucose-1-phosphate thymidylyltransferase
737893	substitution	C	CCCCCCCCCCCCCCCC	CCCCCCCCCCCCCCTCCC	R=>C	M5005_Spy_0735	*cpsFP*	202	68	dTDP-4-dehydrorhamnose 3;5-epimerase
744980	substitution	T	TTTTTTTCTTTTTTTT	TTTTTTTTTTTTTTTTTT	synonymous	M5005_Spy_0743		174	58	ABC transporter substrate-binding protein
755601	substitution	T	TTTTTTTTTTTTTTTT	TTTTTTTTTTTTTTCTTT	synonymous	M5005_Spy_0753	*acoC*	240	80	branched-chain alpha-keto acid dehydrogenase subunit E2
760168	substitution	G	GGGGGGGGGGGGGGGG	GGGTGGGGGGGGGGGGGG	S=>R	M5005_Spy_0757	*hylA*	1785	595	hyaluronate lyase
768320	substitution	C	AAAAAAAAAAAAAAAA	AAAAAAAAAAAAAAAAAA	D=>E	M5005_Spy_0763	*glmM*	939	313	phosphoglucosamine mutase
769429	substitution	G	GGGGGGGGGGGGGGGG	GGGGGAGGGGAGGGGGGG	V=>I	M5005_Spy_0764		559	187	hypothetical protein
774048	substitution	G	AGGGGGGAGAAGGGGG	AAAAGGGAAGGAGGGAAA	G=>S	M5005_Spy_0769		706	236	hypothetical protein
778937	substitution	G	AGGGGGGAGAAGGGGG	AAAAGGGAAGGAGGGAAA	D=>N	M5005_Spy_0772		292	98	hypothetical protein
779476	substitution	A	AAAAAAAAAAAAGAAA	AAAAAAAAAAAAAAAAAA	L=>P	M5005_Spy_0773		167	56	hypothetical protein
779869	substitution	T	TTTTTTTTTTTTTTTT	TTTTCTTTTCTTCCTTTT	L=>P	M5005_Spy_0774		95	32	nucleoside diphosphate kinase
782225	substitution	A	GGGGGGGGGGGGGGGG	GGGGGGGGGGGGGGGGGG						
786972	substitution	C	CCCCCCCCCCCCCCCC	CCCGCCCCCCCCCCCCGC	D=>E	M5005_Spy_0782	*ptsC*	798	266	PTS system mannose/fructose family transporter subunit IIC
790259	substitution	A	AGGAAAAAAAAAAGAA	AAAAAAAAAAAAAAAAAA	synonymous	M5005_Spy_0785		663	221	two-component response regulator
791412	substitution	T	AAAAAAAAAAAAAAAA	AAAAAAAAAAAAAAAAAA	V=>E	M5005_Spy_0786		1034	345	iron(III)-binding protein
801642	substitution	G	GGGGGGGGGGGGGGGG	GGGGGGGGGGGGGGAGGG	V=>I	M5005_Spy_0796	*rplL*	115	39	50S ribosomal protein L7/L12
807991	substitution	T	TTTTTTTTTTTTTTTT	TTTCTTTTTTTTTTTTCT						
812411	substitution	T	TCCTTTCTTTTTCCTT	TTTTTTTTTTTTTTTTTT	I=>T	M5005_Spy_0817	*dacA1*	38	13	D-alanyl-D-alanine carboxypeptidase
813925	substitution	G	GGGGGGGGGGGGGGAA	GGGGGGGGGGGGGGGGGG	T=>I	M5005_Spy_0818		857	286	polysaccharide deacetylase
819178	substitution	C	CCCCCCCCCCCCCCCC	CCCTCCCCCCCCCCCCTC	synonymous	M5005_Spy_0825	*murB*	354	118	UDP-N-acetylenolpyruvoylglucosamine reductase
819844	substitution	C	CCCCCCCCCCCCCCCC	TTCCCCCTTCCCCCCCCC	synonymous	M5005_Spy_0826	*potA*	87	29	spermidine/putrescine transporter ATP-binding protein
836872	substitution	T	TTTTTTTTTTTTTTTT	TTTTCTTTTTTTTTTTTT	D=>G	M5005_Spy_0843		161	54	hypothetical protein
848357	substitution	A	AGGAAAAAAAAAAGAA	AAAAAAAAAAAAAAAAAA	T=>A	M5005_Spy_0857	*guaC*	316	106	guanosine 5'-monophosphate oxidoreductase
850215	substitution	A	TTTTTTTTTTTTTTTT	TTTTTTTTTTTTTTTTTT	K=>M	M5005_Spy_0859		305	102	xanthine permease
852270	substitution	A	AAAAAAAAAAAAAAAA	AAAAAAAAAGAAGGAAAA	synonymous	M5005_Spy_0861		165	55	4-oxalocrotonate tautomerase
855905	substitution	C	CCCCCCCCCCCCCCCC	CCCCCCCCCCCCCCTCCC	L=>F	M5005_Spy_0866		220	74	phosphinothricin N-acetyltransferase
857081	substitution	C	CCCCCCCCCCCCCCCC	CCTCCCCCCCCTCCCTCT	synonymous	M5005_Spy_0867	*glyA*	954	318	serine hydroxymethyltransferase
861703	substitution	G	GGGGGGGGGGGGGGGG	GGGGGGGGGAGGAAGGGG	synonymous	M5005_Spy_0871		1005	335	multidrug resistance ABC transporter ATP-binding protein/permease
863385	substitution	A	AGGAAAAAAAAAAGAA	AAAAAAAAAAAAAAAAAA	D=>G	M5005_Spy_0872	*nox*	419	140	NADH oxidase H2O-forming
885506	substitution	G	GGGGGGGGGGGGGGAA	GGGGGGGGGGGGGGGGGG	synonymous	M5005_Spy_0897		480	160	oxaloacetate decarboxylase subunit beta
885552	substitution	G	GGGAGGGGGGGGGGGG	GGGGGGGGGGGGGGGGGG	A=>T	M5005_Spy_0897		526	176	oxaloacetate decarboxylase subunit beta
889743	substitution	G	GGGGGGGGGGGGGGGG	GGGGGGGGGGGGGGGAGG						
893042	substitution	G	GGGGGGGAGGGGGGGG	GGGGGGGGGGGGGGGGGG	A=>T	M5005_Spy_0906	*citE*	718	240	citrate lyase subunit beta/citryl-CoA lyase subunit
894566	substitution	C	CCCCAACCACCACCCC	CCCCCCACCCCCCCCCCC	S=>Y	M5005_Spy_0907	*citF*	1382	461	citrate lyase subunit alpha/citrate CoA-transferase
898181	substitution	G	GGGGGGGGGGGGGGGG	GGGGGTGGGGTGGGGGGG	H=>N	M5005_Spy_0911		250	84	hypothetical protein
903027	substitution	G	GGGGGGGGGGGGGGAA	GGGGGGGGGGGGGGGGGG	T=>I	M5005_Spy_0915	*ffh*	20	7	signal recognition particle subunit FFH/SRP54
903468	substitution	G	GGGGGGGGGGGGGGGG	GGGGGAGGGGAGGGGGGG						
906088	substitution	G	AGGGGGGGGAAGGGGG	AAAAGGGAAGGAGGGAAA	G=>S	M5005_Spy_0919	*guaA*	217	73	GMP synthase
910071	substitution	G	GGGGGGGGGGGGGGGG	GGGGGGGGGGAGGGGGGG	P=>S	M5005_Spy_0921		538	180	ABC transporter ATP-binding protein
925946	substitution	G	GGGGGGGGGGGGGGGG	GGGGAGGGGGGGGGGGGG	A=>T	M5005_Spy_0937		280	94	transporter
928138	substitution	T	TTTTTTTTTTTTTTTT	TTTTCTTTTTTTTTTTTT						
928684	substitution	C	CCCCCCCCCCCCCCCC	CCCCCCCCCCCCCCTCCC	V=>M	M5005_Spy_0939		616	206	nucleoside transporter permease
934861	substitution	T	TTTTTTTTTTTTCTTT	TTTTTTTTTTTTTTTTTT						
936113	substitution	A	AAAAAAAAAAAAAAAA	AAAAAAAAAAAAACAAAA	K=>T	M5005_Spy_0946	*rpsT*	47	16	30S ribosomal protein S20
937343	substitution	T	CCCCCCCCCCCCCCCC	CCCCCCCCCCCCCCCCCC	synonymous	M5005_Spy_0947	*ciaH*	393	131	sensor protein
942133	substitution	G	GGGGGGGGGGGGGGGG	GGGGAGGGGAGGAAGGGG	T=>M	M5005_Spy_0950	*phoU*	11	4	phosphate transporter protein
946740	substitution	T	TTTTTTTTTTTTTTTT	TTTTTTTTTCTTCTTTTT	synonymous	M5005_Spy_0956		1200	400	16S rRNA m(5)C 967 methyltransferase
950772	substitution	C	CCCCCCCCCCCCCCCC	CCCCCCCCCTCCTTCCCC	S=>N	M5005_Spy_0961	*truB*	521	174	tRNA pseudouridine synthase B
957856	substitution	T	CCCCCCCCCCCCCCCC	CCCCCCCCCCCCCCCCCC						
958092	substitution	A	AAAAAAAAAAAAGAAA	AAAAAAAAAAAAAAAAAA	synonymous	M5005_Spy_0968		123	41	TetR family transcriptional regulator
958210	substitution	C	CCCCCCCCCCCCCCCC	TTCCCCCTTCCCCCCCCC	Q=>*	M5005_Spy_0968		241	81	TetR family transcriptional regulator
959316	substitution	C	CCCCCCCCCCCCCCCC	CCCCTCCCCTCCTTCCCC						
965823	substitution	G	GGGAGGGGGGGGGGGG	GGGGGGGGGGGGGGGGGG	S=>N	M5005_Spy_0978		767	256	Na(+)-linked D-alanine glycine permease
968465	substitution	C	TCCCCCCCCCCCCCCC	CCCCCCCCCCCCCCCCCC	G=>R	M5005_Spy_0981	*cfa*	412	138	cAMP factor
969459	substitution	G	GGGGGGGGGGGGGGGG	GGGGGGAGGGGGGGGGGG	synonymous	M5005_Spy_0982		624	208	histidine-binding protein
970946	substitution	C	CCCCCCCCCCCCCCCC	CCCCCCCCCCCCCCTCCC	V=>I	M5005_Spy_0984		433	145	histidine transporter permease
980687	substitution	C	CTTCCCCCCCCCCTCC	CCCCCCCCCCCCCCCCCC						
981034	substitution	C	TCCCCCCCCTTCCCCC	TTTTCCCTTCCTCCCTTT	T=>I	M5005_Spy_0991		302	101	GntR family transcriptional regulator
981045	substitution	T	TTTTTTTTTTTTTTGG	TTTTTTTTTTTTTTTTTT	F=>V	M5005_Spy_0991		313	105	GntR family transcriptional regulator
1019446	substitution	G	GGGAGGGGGGGGGGGG	GGGGGGGGGGGGGGGGGG	S=>N	M5005_Spy_1050		737	246	phage transcriptional repressor
1022830	substitution	C	CCCCCCCCCCCCCCCC	CCCTCCCCCCCCCCCCTC						
1023123	substitution	T	TTTTTTTTTTTTTTTT	TTTTTTTTTCTTCCTTTT	D=>G	M5005_Spy_1055	*glgP*	2054	685	glycogen phosphorylase
1028037	substitution	G	GGGGGGGGGGGGGGGG	GGGGGGGGGAGGAAGGGG	synonymous	M5005_Spy_1058	*malE*	159	53	maltose/maltodextrin-binding protein
1028515	substitution	T	TTTTTTTGTTTTTTTT	TTTTTTTTTTTTTTTTTT	synonymous	M5005_Spy_1058	*malE*	636	212	maltose/maltodextrin-binding protein
1032390	substitution	G	GGGGGGGGGGGGAGGG	GGGGGGGGGGGGGGGGGG	S=>N	M5005_Spy_1061		434	145	LacI family transcriptional regulator
1038781	substitution	C	CCCCCCCCCCCCTCCC	CCCCCCCCCCCCCCCCCC	A=>T	M5005_Spy_1066	*amyB*	1033	345	neopullulanase/cyclomaltodextrinase/maltogenic alpha-amylase
1045796	substitution	C	CCCCCCCCCCCCCCCC	CCCCCCCCCCCCCCTCCC	V=>I	M5005_Spy_1073	*dltA*	1363	455	D-alanine--poly(phosphoribitol) ligase subunit 1
1046966	substitution	T	CCCCCCCCCCCCCCCC	CCCCCCCCCCCCCCCCCC	N=>D	M5005_Spy_1073	*dltA*	193	65	D-alanine--poly(phosphoribitol) ligase subunit 1
1050732	substitution	G	GGGGGGGGGGGGGGGG	TTGGGGGTTGGGGGGGGG	synonymous	M5005_Spy_1076	*glnH*	972	324	transporter
1055827	substitution	C	CAACCCCCCCCCCACC	CCCCCCCCCCCCCCCCCC	synonymous	M5005_Spy_1083		1815	605	PTS system; mannitol (cryptic)-specific IIA component
1057620	substitution	C	CCCCCCTCCCCCCCCC	CCCCCCCCCCCCCCCCCC	A=>T	M5005_Spy_1083		22	8	PTS system; mannitol (cryptic)-specific IIA component
1064288	substitution	C	CCCCCCCCCCCCTCCC	CCCCCCCCCCCCCCCCCC	synonymous	M5005_Spy_1091		459	153	transposase
1064309	substitution	T	TTTTTTTTTTTTATTT	TTTTTTTTTTTTTTTTTT	D=>E	M5005_Spy_1091		480	160	transposase
1064562	substitution	C	CCCCCCCCCCCCCCCC	CCCCTCCCCTCCTTCCCC	synonymous	M5005_Spy_1092	*rsuA*	708	236	ribosomal small subunit pseudouridine synthase A
1066919	substitution	G	GGGGGGGGGGGGAGGG	GGGGGGGGGGGGGGGGGG	synonymous	M5005_Spy_1094		168	56	major facilitator transporter
1070394	substitution	G	GGGGGGGAGGGGGGGG	GGGGGGGGGGGGGGGGGG	T=>M	M5005_Spy_1098		1316	439	tRNA (uracil-5-)-methyltransferase
1075691	substitution	T	TTTTTTTTTTTTCTTT	TTTTTTTTTTTTTTTTTT	synonymous	M5005_Spy_1102		603	201	ribonuclease BN
1079996	substitution	G	GGGGGGGGGGGGGGGG	GGGGGGGGGGGGGCGGGG						
1083883	substitution	C	CCCCCCCCCCCCCCCC	CCCCCCCCCCCCCCTCCC	G=>E	M5005_Spy_1109	*inlA*	1916	639	internalin protein
1090446	substitution	T	TTTTTTCTTTTTTTTT	TTTTTTTTTTTTTTTTTT	T=>A	M5005_Spy_1115		208	70	hypothetical protein
1090686	substitution	C	CCCCCCCCCCCCCCCC	CCCCCCCCCACCAACCCC						
1090730	substitution	C	CCCCCCCCCCCCCCCC	CCCCCCCCCCCCCCTCCC						
1091220	substitution	G	GGGGGGGGGGGGGGGG	GGGGGAGGGGAGGGGGGG	S=>F	M5005_Spy_1116	*udk*	140	47	uridine kinase
1098241	substitution	A	AAAAAAAAAAAGAAAA	AAAAAAAAAAAAAAAAAA	I=>V	M5005_Spy_1122	*nrdH*	214	72	glutaredoxin
1100892	substitution	C	CCCCTTCCCCCCCCCC	CCCCCCTCCCCCCCCCCC	synonymous	M5005_Spy_1124	*nrdF*	135	45	ribonucleotide-diphosphate reductase subunit beta
1103478	substitution	C	CCCCCCCCCCCTCCCC	CCCCCCCCCCCCCCCCCC	H=>Y	M5005_Spy_1127		10	4	transposase pseudogene
1104077	substitution	T	TTTTTTTTTTTTTTTT	TTTGTTTTTTTTTTTTGT						
1104894	substitution	G	GGGGGGGGGGGGGGGG	GGAGGGGGGGGAGGGAGG	Q=>*	M5005_Spy_1129		193	65	CAAX amino protease
1105713	substitution	T	CTTTTTTCTCCTTTCC	CCCCTTTCCTTCTTTCCC	N=>D	M5005_Spy_1130		154	52	hypothetical protein
1106359	substitution	G	GGGGGGGTGGGGGGGG	GGGGGGGGGGGGGGGGGG						
1110347	substitution	G	GGGGGGGGGGGGGGGG	GGGGGAGGGGAGGGGGGG	synonymous	M5005_Spy_1133	*prsA*	153	51	foldase PrsA

1112101	substitution	C	CTCCCCCCCCCCCCCC	CCCCCCCCCCCCCCCCCC	G=>D	M5005_Spy_1135		431	144	oxalate/formate antiporter
1120139	substitution	G	GAAGGGGGGGGGGAGG	GGGGGGGGGGGGGGGGGG	P=>S	M5005_Spy_1142		544	182	hypothetical protein
1120994	substitution	T	TTTTTTTTTTTTTTTT	TTTTCTTTTCTTCCTTTT	K=>R	M5005_Spy_1144		146	49	hypothetical protein
1129287	substitution	T	TTTTTTGTTTTTTTTT	TTTTTTTTTTTTTTTTTT	K=>N	M5005_Spy_1153		1779	593	kup system potassium uptake protein
1144824	substitution	C	ACCCCCCCCAACCCCC	AAAACCCAACCACCCAAA	M=>I	M5005_Spy_1167		318	106	lead; cadmium; zinc and mercury transporting ATPase
1144936	substitution	T	TTTTTTTTTTTTTTGG	TTTTTTTTTTTTTTTTTT	D=>A	M5005_Spy_1167		206	69	lead; cadmium; zinc and mercury transporting ATPase
1180290	substitution	A	AAAAAAAAAGGAAAAA	AAAAAAAAAAAAAAAAAA	synonymous	M5005_Spy_1225		375	125	lipase/acylhydrolase
1181323	substitution	G	GGGGGGGGGGGGGGGG	GGGGGGGGGGGGGGGGGA	synonymous	M5005_Spy_1226		186	62	degV family protein
1183695	substitution	C	CCCCCCCCCCCCCCCC	CCCTCCCCCCCCCCCCCC	synonymous	M5005_Spy_1228	*recN*	804	268	DNA repair protein
1186376	substitution	T	TTTTTTTTTTTTTTTT	TTTTTTTTTTTTTTCTTT	synonymous	M5005_Spy_1231	*fps*	294	98	dimethylallyltransferase/geranyltranstransferase
1190164	substitution	G	AGGGGGGGGGGGGGGG	AAGAGGGAAGGGGGGGAG	synonymous	M5005_Spy_1235		957	319	phosphoglucomutase
1190317	substitution	G	GGGGGGGGGTTGGGGG	GGGGGGGGGGGGGGGGGG	synonymous	M5005_Spy_1235		804	268	phosphoglucomutase
1193974	substitution	A	AAAAAAAGAAAAAAAA	AAAAAAAAAAAAAAAAAA	synonymous	M5005_Spy_1238	*artQ*	294	98	arginine transporter permease
1195199	substitution	G	GGGGGGGGGTTGGGGG	GGGGGGGGGGGGGGGGGG	G=>V	M5005_Spy_1240	*clpE*	278	93	ATP-dependent Clp protease ATP-binding subunit
1201647	substitution	A	AAAAAAAAAAAAAAAA	AAAAAAAAACAACCAAAA	synonymous	M5005_Spy_1244	*divIVAS*	591	197	cell division initiation protein
1208561	substitution	G	GGGTGGGGGGGGGGGG	GGGGGGGGGGGGGGGGGG	A=>X	M5005_Spy_1251	*divIB*	146	49	cell division protein
1212071	substitution	G	GGGGGGGGGGGGGGGG	GGGGAGGGGAGGAAGGGG	A=>V	M5005_Spy_1255	*typA*	1661	554	GTP-binding protein
1215160	substitution	T	TTTTTTTTTTTTTTTT	TTTTTCTTTTCTTTTTTT	H=>R	M5005_Spy_1257	*glcK*	125	42	glucokinase/xylose repressor
1219311	substitution	C	CGGCCCCCCCCCCGCC	CCCCCCCCCCCCCCCCCC	synonymous	M5005_Spy_1264		639	213	ribose operon repressor
1219760	substitution	T	CCCCCCCCCCCCCCCC	CCCCCCCCCCCCCCCCCC	T=>A	M5005_Spy_1264		190	64	ribose operon repressor
1227170	substitution	C	CCCCTCCCCCCCCCCC	CCCCCCCCCCCCCCCCCC	G=>E	M5005_Spy_1272		752	251	arginine/ornithine antiporter
1230233	substitution	A	AAAAAAAAAAAAAAAA	AAAAAAAAACAACCAAAA	synonymous	M5005_Spy_1275	*arcA*	675	225	arginine deiminase
1233524	substitution	A	GGGGGGGGGGGGGGGG	GGGGGGGGGGGGGGGGGG	C=>R	M5005_Spy_1279		928	310	hypothetical protein
1233694	substitution	G	GGGGGGGGGGGGGGGG	GGGGGGGGGGGGGGAGGG	A=>V	M5005_Spy_1279		758	253	hypothetical protein
1238880	substitution	T	TCCTTTTTTTTTTCTT	TTTTTTTTTTTTTTTTTT	Q=>R	M5005_Spy_1284	*ccdA*	698	233	cytochrome C biogenesis protein
1241140	substitution	G	GGGGGGGAGGGGGGGG	GGGGGGGGGGGGGGGGGG	R=>C	M5005_Spy_1286		370	124	DNA polymerase
1241346	substitution	G	GGGGGGGGGGGGGGGG	GGGGGGGGGAGGAAGGGG	A=>V	M5005_Spy_1286		164	55	DNA polymerase
1242500	substitution	G	GGGGGGGGGGGGGGGG	TTGGGGGGTGGGGGGGGG	synonymous	M5005_Spy_1288		531	177	hypothetical protein
1245491	substitution	G	GGGGGGGGGGGGGGGG	GGGGGGAGGGGGGGGGGG	synonymous	M5005_Spy_1290		168	56	hypothetical protein
1246611	substitution	C	CTTCCCCCCCCCCTCC	CCCCCCCCCCCCCCCCCC	R=>K	M5005_Spy_1291		1583	528	ATP-dependent RNA helicase
1249539	substitution	G	GAAGGGAGGGGGAAGG	GGGGGGGGGGGGGGGGGG	S=>L	M5005_Spy_1292	*valS*	1220	407	valyl-tRNA synthetase
1255457	substitution	G	GGGGGGGGGGGGGGGG	GGGGAGGGGGGGGGGGGG						
1258675	substitution	C	CCCCCCCCCCCCCCTT	CCCCCCCCCCCCCCCCCC	synonymous	M5005_Spy_1304	*lacZ*	3075	1025	beta-galactosidase
1259620	substitution	A	AAAAAAAAAAAAAAAA	AAAAAAAAAAAAAAGAAA	synonymous	M5005_Spy_1304	*lacZ*	2130	710	beta-galactosidase
1266055	substitution	C	CCCCCCCCCCCCTCCC	CCCCCCCCCCCCCCCCCC	synonymous	M5005_Spy_1308		1269	423	sugar-binding protein
1269730	substitution	A	AAGAAAAAAAAAAGAA	AAAAAAAAAAAAAAAAAA	H=>R	M5005_Spy_1311		245	82	glucokinase
1271787	substitution	G	GGGGGGGGAGGGGGGG	GGGGGGGGGGGGGGGGGG	P=>S	M5005_Spy_1313		631	211	beta-glucosidase
1271817	substitution	C	CCCCCCCCCCCCCCCC	CCCCCCCCCCCCCCTCCC	A=>T	M5005_Spy_1313		601	201	beta-glucosidase
1275340	substitution	T	TTTTTTTTTTTTCTTT	TTTTTTTTTTTTTTTTTT						
1277466	substitution	A	AAAACCAACAACAAAA	AAAAAACAAAAAAAAAAA	synonymous	M5005_Spy_1317		807	269	alpha-mannosidase
1291304	substitution	A	AAAAGGAAGAAGAAAA	AAAAAAGAAAAAAAAAAA	Y=>C	M5005_Spy_1324		59	20	hypothetical protein
1294405	substitution	G	GGGGGGGGGGGGGGGG	GGGGGGGGGGGGGGAGGG	L=>F	M5005_Spy_1327	*comFA*	112	38	competence protein ComF
1295506	substitution	C	CCCCAACCACCACCCC	CCCCCCACCCCCCCCCCC	R=>S	M5005_Spy_1329	*cysM*	64	22	cysteine synthase
1297904	substitution	C	CCCCCCCCCCCCCCCC	CCCCCCTCCCCCCCCCCC	synonymous	M5005_Spy_1331		276	92	peptidyl-prolyl cis-trans isomerase
1300980	substitution	A	AAAAAAAAAAAAAAAA	AAAAAAAAAAAAAAAAAG	synonymous	M5005_Spy_1335		1585	529	serine/threonine protein kinase
1310853	substitution	T	ATTTTTTTTAATTTTT	AAAATTTAATTATTTAAA						
1310932	substitution	G	GGGGGGGGGGGAGGGG	GGGGGGGGGGGGGGGGGG	synonymous	M5005_Spy_1343		877	293	LysR family transcriptional regulator
1315198	substitution	G	GGGGGGGGGGGGGGGG	GGGGGGGGGAGGAAGGGG	E=>K	M5005_Spy_1347		634	212	3-hydroxybutyrate dehydrogenase
1317981	substitution	C	CCCCTTCCTCCTCCCC	CCCCCCTCCCCCCCCCCC	synonymous	M5005_Spy_1350		936	312	hypothetical protein
1324752	substitution	G	GGGGGGGGGGGGGGGG	AAGGGGGAAGGGGGGGGG						
1334794	substitution	G	GGGGGGAGGGGGGGGG	GGGGGGGGGGGGGGGGGG						
1346645	substitution	G	GGGGGGGGGGGGGGAA	GGGGGGGGGGGGGGGGGG	synonymous	M5005_Spy_1375	*tkt*	279	93	transketolase
1350781	substitution	G	GGGGGGGGGGGGGGGG	GGGGAGGGGGGGGGGGGG						
1357909	substitution	G	GGGGGGGGGGGGGAGG	GGGGGGGGGGGGGGGGGG	synonymous	M5005_Spy_1384	*glyS*	360	120	glycyl-tRNA synthetase subunit beta
1358964	substitution	A	AAAAAAAAAAAAAAAA	AAAAAAAAAAAAAACAAA	H=>Q	M5005_Spy_1385	*glyQ*	600	200	glycyl-tRNA synthetase subunit alpha
1366216	substitution	A	AAAAAAAAAAAAAAAA	AAAGAAAAAAAAAAAAGA	synonymous	M5005_Spy_1391		138	46	degV family protein
1371669	substitution	G	GGGGGGAGGGGGGGGG	GGGGGGGGGGGGGGGGGG	synonymous	M5005_Spy_1399		1341	447	PTS system galactose-specific transporter subunit IIC
1373679	substitution	A	AAAAAAAAAAAAAAAA	AAAAACAAAACAAAAAAA	V=>G	M5005_Spy_1401		131	44	PTS system galactose-specific transporter subunit IIA
1386360	substitution	C	CTCCCCCCCCCCCCCC	CCCCCCCCCCCCCCCCCC	P=>L	M5005_Spy_1415	*sdaD2*	593	198	phage-encoded streptodornase
1386846	substitution	C	CCCCCCCCCCCCCCCC	AACCCCCAACCCCCCCCC	P=>H	M5005_Spy_1415	*sdaD2*	1079	360	phage-encoded streptodornase
1391304	substitution	A	CCCCCCCCCCCCCCCC	CCCCCCCCCCCCCCCCCC	synonymous	M5005_Spy_1421		252	84	phage infection protein
1398809	substitution	G	GGGGGGGGGGGGGGAA	GGGGGGGGGGGGGGGGGG	A=>V	M5005_Spy_1426		1673	558	phage protein
1400357	substitution	G	GGGGGGGGGGGGGGGG	GGGGGGGGGGGGGGGGGC	A=>G	M5005_Spy_1426		125	42	phage protein
1411918	substitution	C	CCCCCCCCCCCCCCCC	CCCCCCCCCTCCTTCCCC	A=>T	M5005_Spy_1446		127	43	phage protein
1414114	substitution	C	CCCCCCCCCCCCTCCC	CCCCCCCCCCCCCCCCCC	R=>K	M5005_Spy_1449		2297	766	DNA primase
1423327	substitution	A	AAAAAGAAAAAAAAAA	AAAAAAAAAAAAAAAAAA						
1424174	substitution	T	TCCTTTTTTTTTTCTT	TTTTTTTTTTTTTTTTTT	M=>T	M5005_Spy_1465		350	117	phage protein
1425135	substitution	G	GGGGGGGGGGGGGGGG	GGGGGGGGGGGGGGAGGG	synonymous	M5005_Spy_1467	*int.3*	249	83	integrase
1425540	substitution	T	TTTTTTTTTTTTTTTT	TTTTTTTTTATTAATTTT	N=>K	M5005_Spy_1467	*int.3*	654	218	integrase
1444665	substitution	G	GGGGGGGGGGGGGGGG	GGGGGAGGGGAGGGGGGG	S=>L	M5005_Spy_1488	*accB*	131	44	acetyl-CoA carboxylase biotin carboxyl carrier protein subunit
1446893	substitution	C	CTTCCCCCCCCCCTCC	CCCCCCCCCCCCCCCCCC	V=>I	M5005_Spy_1491	*fabD*	814	272	ACP S-malonyltransferase
1457677	substitution	C	CCCCCCCCCCCCCCCC	CCCCCCCCCCCCCCTCCC	D=>N	M5005_Spy_1502		508	170	D-alanyl-D-alanine carboxypeptidase
1458319	substitution	A	GGGGGGGGGGGGGGGG	GGGGGGGGGGGGGGGGGG	L=>P	M5005_Spy_1503		563	188	phosphoglycerate mutase
1459080	substitution	A	AAAAAAAAAAAAAAAA	AAAAAAAAAGAAGAAAAA						
1459409	substitution	G	AGGAAAGAAAAAGGAA	AAAAGGAAAGGAGGAAAA	L=>F	M5005_Spy_1504		112	38	hypothetical protein
1467375	substitution	T	CCCCCCCCCCCCCCCC	CCCCCCCCCCCCCCCCCC	V=>A	M5005_Spy_1514		86	29	universal stress protein
1486375	substitution	T	TCCTTTCTTTTTCCTT	TTTTTTTTTTTTTTTTTT	N=>D	M5005_Spy_1530		2272	758	Fe3+-siderophore transporter
1488161	substitution	A	GGGGGGGGGGGGGGGG	GGGGGGGGGGGGGGGGGG	synonymous	M5005_Spy_1530		486	162	Fe3+-siderophore transporter
1496036	substitution	G	TGGTTTGTTTTTGGTT	TTTTGGTTTGGTGGTTTT	S=>Y	M5005_Spy_1537		74	25	transposase
1506110	substitution	A	AAAAAAAAAAAACAAA	AAAAAAAAAAAAAAAAAA	D=>A	M5005_Spy_1544	*scrR*	857	286	sucrose operon repressor
1508494	substitution	G	AGGGGGGGGAAGGGGG	AAAAGGGAAGGAGGGAAA	synonymous	M5005_Spy_1549		969	323	Xaa-Pro dipeptidase
1510945	substitution	C	TCCCCCCTCTTCCCCC	TTTTCCCTTCCTCCCTTT	synonymous	M5005_Spy_1550	*uvrA*	1491	497	excinuclease ABC subunit A
1518175	substitution	T	TTTTTTTTTTTTTTTT	TTTTTTTTTCTTCCTTTT						
1518950	substitution	G	GGGGGGGGGGGGGGGG	GGGGGGGGGGGGGGAGGG	synonymous	M5005_Spy_1560		133	45	phosphatidylglycerophosphatase B
1533612	substitution	G	GGGGGGGGGGGGGGGG	GGGGGGAGGGGGGGGGGG	V=>I	M5005_Spy_1572		553	185	hypothetical protein
1535301	substitution	T	TTTTTTCTTTTTTTTT	TTTTTTTTTTTTTTTTTT						
1536100	substitution	C	CCCCCCCCCCCCCCCC	CCCCCCCCCCCCCCCCCT	G=>S	M5005_Spy_1575	*norA*	739	247	quinolone resistance protein
1546294	substitution	C	CCCCCCCCCCCCCCCC	CCCCTCCCCCCCCCCCCC	D=>N	M5005_Spy_1586	*nupC*	493	165	nucleoside permease
1548628	substitution	G	GGGGGGGGGGGGGGGG	GGGGGTGGGGTGGGGGGG						
1553968	substitution	T	TTTTTTTCTTTTTTTT	TTTTTTTTTTTTTTTTTT	N=>S	M5005_Spy_1596	*glnA*	1193	398	glutamine synthetase
1558314	substitution	T	TTTTCCTTTTTTTTTT	TTTTTTTTTTTTTTTTTT	D=>G	M5005_Spy_1600	*lppC*	380	127	acid phosphatase
1568321	substitution	T	TTTTTTTTTTTTTTTT	TTTTCTTTTCTTCCTTTT	synonymous	M5005_Spy_1611	*rpoE*	564	188	DNA-directed RNA polymerase subunit delta
1573757	substitution	G	GGGGGGGGGAAGGGGG	GGGGGGGGGGGGGGGGGG	synonymous	M5005_Spy_1617	*truA*	396	132	tRNA pseudouridine synthase A
1581669	substitution	A	AAAAAGAAAAAAAAAA	AAAAAAAAAAAAAAAAAA	L=>S	M5005_Spy_1620		80	27	glycerate kinase
1587039	substitution	C	CCCCCCCCCCCCCCCC	CCCCCTCCCCTCCCCCCC	synonymous	M5005_Spy_1623	*hsdM*	807	269	type I restriction-modification system methylation subunit
1595156	substitution	G	GGGGAAGGAGGAGGGG	GGGGGGAGGGGGGGGGGG	synonymous	M5005_Spy_1630	*salB*	202	68	serine (threonine) dehydratase
1595227	substitution	G	AAAAAAAAAAAAAAAA	AAAAGGAAAGGAGGAAAA	T=>I	M5005_Spy_1630	*salB*	131	44	serine (threonine) dehydratase
1595847	substitution	A	AAAAAAAAAAAAGAAA	AAAAAAAAAAAAAAAAAA						
1598629	substitution	C	CCCCCCCCCCCCCCCC	CCCCCCCCCTCCTTCCCC	D=>N	M5005_Spy_1633	*lacE*	661	221	PTS system lactose-specific transporter subunit IIBC
1607118	substitution	C	TTTTTTTTTTTTTTTT	TTTTCCTTTCCTCCTTTT	G=>D	M5005_Spy_1647	*rplM*	32	11	50S ribosomal protein L13
1612779	substitution	G	GGGGGGGGAGGGGGGG	GGGGGGGGGGGGGGGGGG	H=>Y	M5005_Spy_1655	*cysS*	448	150	cysteinyl-tRNA synthetase
1617521	substitution	C	CCCCCCCCCCCCCCCC	CCCCCCCCCCCCCCCTCC						
1622441	substitution	A	AAAAAAAAAAAAAAAA	AAAAATAAAATAAAAAAA	L=>*	M5005_Spy_1665		122	41	hypothetical protein
1622460	substitution	A	AAAAAAAAAAAAAAAA	AAAAAGAAAAGAAAAAAA	W=>R	M5005_Spy_1665		103	35	hypothetical protein
1623818	substitution	T	TTTTTTTTTTTTTTTT	TTTTTTTTTTTTTTTTTC	synonymous	M5005_Spy_1669	*def*	195	65	peptide deformylase
1624302	substitution	A	AAAAAAAAAAAACAAA	AAAAAAAAAAAAAAAAAA	synonymous	M5005_Spy_1670		513	171	oxidoreductase
1624692	substitution	A	GGGGGGGGGGGGGGGG	GGGGGGGGGGGGGGGGGG	synonymous	M5005_Spy_1670		123	41	oxidoreductase
1629943	substitution	G	GGGGGGGGGGGGGGGG	GGGGGTGGGGTGGGGGGG						
1636821	substitution	A	GGGGGGGGGGGGGGGG	GGGGGGGGGGGGGGGGGG	synonymous	M5005_Spy_1679	*pulA*	2088	696	pullulanase
1637310	substitution	A	ATAAAAAAAAAAAAAA	AAAAAAAAAAAAAAAAAA	N=>K	M5005_Spy_1679	*pulA*	1599	533	pullulanase
1638625	substitution	A	AAAAAAAGAAAAAAAA	AAAAAAAAAAAAAAAAAA	V=>A	M5005_Spy_1679	*pulA*	284	95	pullulanase
1645590	substitution	G	GGGGGGGGGGGGAGGG	GGGGGGGGGGGGGGGGGG	E=>K	M5005_Spy_1684	*ska*	1249	417	streptokinase
1648982	substitution	T	TTTTTTTTTTTTTTTT	TTTTTATTTTATTTTTTT	Q=>M	M5005_Spy_1689	*sclA*	542	181	hypothetical protein
1648983	substitution	G	GGGGGGGGGGGGGGGG	GGGGGTGGGGTGGGGGGG	Q=>M	M5005_Spy_1689	*sclA*	541	181	hypothetical protein
1648984	substitution	G	GGGGGGGGGGGGGGGG	GGGGGCGGGGCGGGGGGG	N=>K	M5005_Spy_1689	*sclA*	540	180	hypothetical protein
1648987	substitution	G	GGGGGGGGGGGGGGGG	GGGGGTGGGGTGGGGGGG	A=>V	M5005_Spy_1689	*sclA*	537	179	hypothetical protein
1648988	substitution	G	GGGGGGGGGGGGGGGG	GGGGGAGGGGAGGGGGGG	A=>V	M5005_Spy_1689	*sclA*	536	179	hypothetical protein
1648991	substitution	T	TTTTTTTTTTTTTTTT	TTTTTATTTTATTTTTTT	Q=>L	M5005_Spy_1689	*sclA*	533	178	hypothetical protein
1659463	substitution	A	AAAAAAAAAAAAGAAA	AAAAAAAAAAAAAAAAAA	synonymous	M5005_Spy_1699		192	64	recombination factor protein RarA
1663603	substitution	T	TTTTTTTCTTTTTTTT	TTTTTTTTTTTTTTTTTT						
1664320	substitution	G	GGGGGGGGGGGGGGGG	GGGGGAGGGGAGGGGGGG	A=>T	M5005_Spy_1704	*dppA*	604	202	dipeptide-binding protein
1664913	substitution	G	GGGGGGGGGGGGGGGG	GGGGGGGGGAGGAAGGGG	synonymous	M5005_Spy_1704	*dppA*	1197	399	dipeptide-binding protein
1665139	substitution	G	GGGGGGGGGGGGGGGG	GGGGGAGGGGAGGGGGGG	E=>K	M5005_Spy_1704	*dppA*	1423	475	dipeptide-binding protein
1669247	substitution	G	GGGGGGGGGGGGGGGG	GGGGGTGGGGTGGGGGGG	Q=>K	M5005_Spy_1710		2359	787	histidine triad protein
1672157	substitution	C	CCCCCCCCCCCCCCCC	CCCCCCCCCCCCCCTCCC	E=>K	M5005_Spy_1711	*lmb*	382	128	laminin binding protein
1674753	substitution	G	GGGGGGGGGGGGGGGG	GGGGGAGGGGAGGGGGGG	P=>S	M5005_Spy_1714		415	139	cell surface protein
1680734	substitution	G	GAAGGGGGGGGGGAGG	GGGGGGGGGGGGGGGGGG						
1680736	substitution	T	TAATTTTTTTTTTATT	TTTTTTTTTTTTTTTTTT						

1681205	substitution	T	T T T T T T T T T T T T T T C C	T T T T T T T T T T T T T T T T T T	D=>G	M5005_Spy_1718	*sic1.01*	713	238	inhibitor of complement protein
1681698	substitution	G	G G G G G G G G G G G G G G G G	G G G G G G G G G G G G G G T G G G	Q=>K	M5005_Spy_1718	*sic1.01*	220	74	inhibitor of complement protein
1683081	substitution	A	A A A A A A A A A A A A G A A A	A A A A A A A A A A A A A A A A A A	A=>V	M5005_Spy_1719	*emm1.0*	480	160	M protein
1683082	substitution	G	G G G G G G G G G G G G A G G G	G G G G G G G G G G G G G G G G G G	A=>V	M5005_Spy_1719	*emm1.0*	479	160	M protein
1683085	substitution	G	G G G G G G G G G G G G T G G G	G G G G G G G G G G G G G G G G G G	T=>N	M5005_Spy_1719	*emm1.0*	476	159	M protein
1683095	substitution	G	G G G G G G G G G G G G T G G G	G G G G G G G G G G G G G G G G G G	H=>N	M5005_Spy_1719	*emm1.0*	466	156	M protein
1683096	substitution	G	G G G G G G G G G G G G A G G G	G G G G G G G G G G G G G G G G G G	synonymous	M5005_Spy_1719	*emm1.0*	465	155	M protein
1683103	substitution	C	C C C C C C C C C C C C T C C C	C C C C C C C C C C C C C C C C C C	R=>Q	M5005_Spy_1719	*emm1.0*	458	153	M protein
1683108	substitution	T	T T T T T T T T T T T T C T T T	T T T T T T T T T T T T T T T T T T	synonymous	M5005_Spy_1719	*emm1.0*	453	151	M protein
1683111	substitution	C	C C C C C C C C C C C C T C C C	C C C C C C C C C C C C C C C C C C	synonymous	M5005_Spy_1719	*emm1.0*	450	150	M protein
1683137	substitution	C	C C C C C C C C C C C C T C C C	C C C C C C C C C C C C C C C C C C	E=>K	M5005_Spy_1719	*emm1.0*	424	142	M protein
1683241	substitution	T	T T T T T T A T T T T T T T T T	T T T T T T T T T T T T T T T T T T	D=>V	M5005_Spy_1719	*emm1.0*	320	107	M protein
1683359	substitution	G	G G G G G G G G A G G A G G G G	G G G G G G G G G G G G G G G G G G	H=>Y	M5005_Spy_1719	*emm1.0*	202	68	M protein
1683380	substitution	T	T T T T T T T T T T T T T T T T	T T T T C T T T T T T T T T T T T T	I=>V	M5005_Spy_1719	*emm1.0*	181	61	M protein
1683390	substitution	G	G G G G G G G G G G G G G G C C	G G G G G G G G G G G G G G G G G G	N=>K	M5005_Spy_1719	*emm1.0*	171	57	M protein
1683392	substitution	T	T C C T T T T T T T T T T C T T	T T T T T T T T T T T T T T T T T T	N=>D	M5005_Spy_1719	*emm1.0*	169	57	M protein
1698040	substitution	G	G G G G G G G G G G G G G G G G	A A G G G G G A A G G G G G G G G G	A=>V	M5005_Spy_1735	*speB*	113	38	exotoxin B
1699488	substitution	T	T T T T T T T T T T T T T T T T	T T T T T T T T T C T T C T T T T T	S=>P	M5005_Spy_1737	*rgg*	397	133	transcriptional regulator
1699903	substitution	A	A A A A A A A A A G G A A A A A	A A A A A A A A A A A A A A A A A A	Y=>C	M5005_Spy_1737	*rgg*	812	271	transcriptional regulator
1707647	substitution	A	G A A A A A A A A G G A A A A A	G G G G A A A G G A A G A A A G G G	M=>T	M5005_Spy_1744		56	19	PTS system cellobiose-specific transporter subunit IIC
1712330	substitution	G	G G G G G G G G G G G G G G G G	G G G A G G G G G G G G G G G G A G	synonymous	M5005_Spy_1753	*pbp2A*	1932	644	multimodular transpeptidase-transglycosylase
1715248	substitution	G	C G G G G G G G G G G G G G G G	C C G C G G G C C G G G G G G G C G	E=>D	M5005_Spy_1755		78	26	hypothetical protein
1716927	substitution	T	T T T T T T T T T T T T T T T T	T T T T T T T T T T T T T T C T T T	T=>A	M5005_Spy_1757		1060	354	hypothetical protein
1732440	substitution	C	T T T T T T T T T T T T T T T T	T T T T T T T T T T T T T T T T T T	synonymous	M5005_Spy_1771	*hutU*	21	7	urocanate hydratase
1734351	substitution	T	T T T T T T T T T T T T T T T T	T T T T T G T T T T G T T T T T T T	I=>M	M5005_Spy_1771	*hutU*	1932	644	urocanate hydratase
1757986	substitution	G	G A G G G G G G G G G G G G G G	G G G G G G G G G G G G G G G G G G	A=>V	M5005_Spy_1789	*nrdG*	170	57	anaerobic ribonucleoside-triphosphate reductase activating protein
1759256	substitution	G	G G G G G G G G G G G G G G G G	G G G G G G G G G G G G G G A G G G	A=>V	M5005_Spy_1791		353	118	virulence factor
1765312	substitution	G	G G G G G G G G A G G G G G G G	G G G G G G G G G G G G G G G G G G						
1766142	substitution	A	A A A A A A A A C A A A A A A A	A A A A A A A A A A A A A A A A A A	F=>C	M5005_Spy_1799	*recA*	1031	344	recombinase A
1792436	substitution	A	A A A A A A A A A A A A A A A A	A A A A A C A A A A C A A A A A A A	T=>P	M5005_Spy_1826		4	2	hypothetical protein
1796058	substitution	T	T T T T T T T T T T T T T T T T	T T G T T T T T T T T G T T T G T G	N=>T	M5005_Spy_1829		56	19	phage infection protein
1797521	substitution	C	C G G C C C C C C C C C C G C C	C C C C C C C C C C C C C C C C C C	V=>L	M5005_Spy_1831	*rpsD*	400	134	30S ribosomal protein S4
1806134	substitution	C	C C C C C C C C C C C C C C C C	T T C C C C C C T C C C C C C C C C						
1809725	substitution	T	C C C C C C C C C C C C C C C C	C C C C C C C C C C C C C C C C C C	synonymous	M5005_Spy_1842	*sdhA*	330	110	L-serine dehydratase
1814690	substitution	C	C C C T C C C C C C C C C C C C	C C C C C C C C C C C C C C C C C C	synonymous	M5005_Spy_1848		1011	337	hypothetical protein
1815161	substitution	G	G G G G G G G G G G G A G G G G	G G G G G G G G G G G G G G G G G G	synonymous	M5005_Spy_1848		540	180	hypothetical protein
1818669	substitution	C	C C C T C C C C C C C C C C C C	C C C C C C C C C C C C C C C C C C						
1821224	substitution	G	A G G G G G G G G A A G G G G G	A A A A G G G A A G G A G G G A A A						
1831530	substitution	T	T T T T T T T T T T T T T T T T	T T T T T C T T T T C T T T T T T T	synonymous	M5005_Spy_1862		384	128	ABC transporter permease
1833384	substitution	A	A A A G A A A A A A A A A A A A	A A A A A A A A A A A A A A A A A A	synonymous	M5005_Spy_1862		2238	746	ABC transporter permease
1835298	substitution	C	C C C C C C C C C C C C C C C C	C C T C C C C C C C C T C C C T C C						

LEGEND

Reference base

Polymorphism

Non-synonymous change

Supplementary Table 3. Distribution of GAS *emm* types from clinical cases presenting at Queen Mary Hospital, Hong Kong (2011-2014).

***emm* type**	**2011**		**2012**		**2013**		**2014 (Jan-Oct)**	
	**Cases**	**%**	**Cases**	**%**	**Cases**	**%**	**Cases**	**%**
*emm*12	44	77.2	44	55	13	31	6	24
*emm*1	6	10.5	13	16.3	11	26.2	13	52
*emm*89	2	3.5	0	0	5	11.9	1	4
*emm*11	1	1.8	0	0	1	2.4	0	0
*emm*22	1	1.8	2	2.5	1	2.4	0	0
*emm*66	1	1.8	0	0	0	0	0	0
*emm*75	1	1.8	0	0	1	2.4	0	0
*emm*79	1	1.8	0	0	0	0	0	0
*emm*2	0	0	1	1.3	0	0	1	4
*emm*3	0	0	1	1.3	0	0	0	0
*emm*4	0	0	2	2.5	1	2.4	0	0
*emm*8	0	0	1	1.3	0	0	0	0
*emm*13L	0	0	1	1.3	0	0	0	0
*emm*28	0	0	0	0	0	0	1	4
*emm*44	0	0	1	1.3	0	0	0	0
*emm*49	0	0	1	1.3	0	0	0	0
*emm*58	0	0	3	3.8	2	4.8	0	0
*emm*63	0	0	0	0	1	2.4	0	0
*emm*67	0	0	1	1.3	0	0	0	0
*emm*68	0	0	1	1.3	0	0	0	0
*emm*74	0	0	1	1.3	0	0	0	0
*emm*76	0	0	0	0	1	2.4	0	0
*emm*77	0	0	1	1.3	1	2.4	1	4
*emm*82	0	0	0	0	2	4.8	0	0
*emm*87	0	0	0	0	1	2.4	0	0
*emm*90	0	0	0	0	0	0	1	4
*emm*91	0	0	0	0	0	0	1	4
*emm*92	0	0	1	1.3	0	0	0	0
*emm*102	0	0	1	1.3	0	0	0	0
*emm*104	0	0	1	1.3	0	0	0	0
*emm*106	0	0	1	1.3	0	0	0	0
*emm*110	0	0	1	1.3	0	0	0	0
*emm*183	0	0	0	0	1	2.4	0	0
Untypable	0	0	1	1.3	0	0	0	0
**Total**	**57**	**100**	**80**	**100**	**42**	**100**	**25**	**100**
